# CAR T cells, CAR NK cells, and CAR macrophages exhibit distinct traits in glioma models but are similarly enhanced when combined with cytokines

**DOI:** 10.1016/j.xcrm.2025.101931

**Published:** 2025-01-30

**Authors:** Thomas Look, Roman Sankowski, Manon Bouzereau, Serena Fazio, Miaomiao Sun, Alicia Buck, Niklas Binder, Maximilian Mastall, Francesco Prisco, Frauke Seehusen, Julia Frei, Conrad Wyss, Berend Snijder, Cesar Nombela Arrieta, Michael Weller, Steve Pascolo, Tobias Weiss

**Affiliations:** 1 Department of Neurology, Clinical Neuroscience Center, University Hospital and University of Zurich, 8091 Zurich, Switzerland; 2 Institute of Neuropathology, Faculty of Medicine, University of Freiburg, 79106 Freiburg, Germany; 3 Department of Medical Oncology and Hematology, University of Zurich and University Hospital Zurich, 8091 Zurich, Switzerland; 4 Laboratory for Animal Model Pathology, Institute of Veterinary Pathology, Vetsuisse Faculty, University of Zurich, 8057 Zurich, Switzerland; 5 Department of Dermatology, University of Zurich and University Hospital Zurich, 8091 Zurich, Switzerland; 6 Institute of Molecular Systems Biology, Department of Biology, ETH Zurich, 8093 Zurich, Switzerland

**Keywords:** glioblastoma, immune regulation, CAR immune cells, tumor homing, single-cell sequencing, preclinical studies, mRNA transfection

## Abstract

Chimeric antigen receptor (CAR) T cell therapy is a promising immunotherapy against cancer. Although there is a growing interest in other cell types, a comparison of CAR immune effector cells in challenging solid tumor contexts is lacking. Here, we compare mouse and human NKG2D-CAR-expressing T cells, natural killer (NK) cells, and macrophages against glioblastoma, the most aggressive primary brain tumor. *In vitro* we show that T cell cancer killing is CAR dependent, whereas intrinsic cytotoxicity overrules CAR dependence for NK cells, and CAR macrophages reduce glioma cells in co-culture assays. In orthotopic immunocompetent glioma mouse models, systemically administered CAR T cells demonstrate superior accumulation in the tumor, and each immune cell type induces distinct changes in the tumor microenvironment. An otherwise low therapeutic efficacy is significantly enhanced by co-expression of pro-inflammatory cytokines in all CAR immune effector cells, underscoring the necessity for multifaceted cell engineering strategies to overcome the immunosuppressive solid tumor microenvironment.

## Highlights

• CAR T cells, CAR NK cells, and CAR macrophages can be efficiently generated with mRNA
• The CAR effector cells exhibit different anti-tumor activities in glioma mouse models
• Each CAR immune cell type has a distinct effect on the tumor microenvironment
• Co-expression of cytokines increases the anti-tumor potential of CAR immune cells

## Graphical abstract

**Figure:**
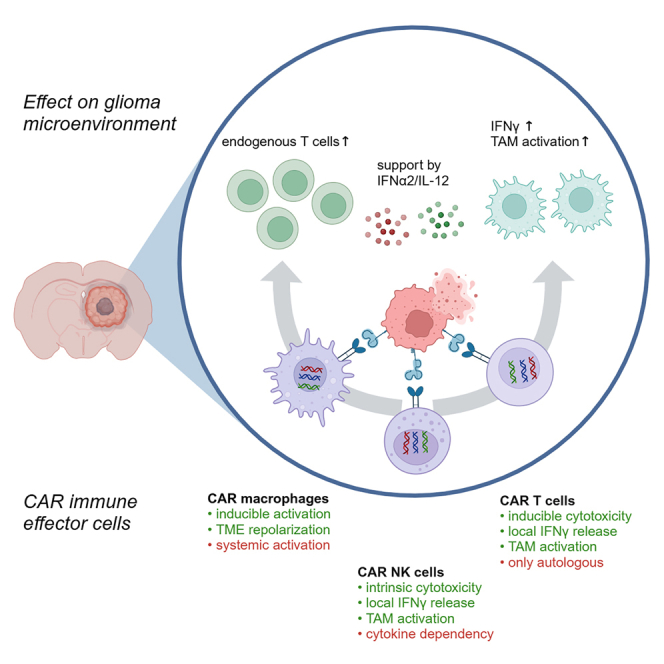

## Teaser

This comparative analysis of CAR T cells, CAR NK cells, and CAR macrophages in glioma mouse models by Look et al. reveals distinct features for each cell type, emphasizes the benefit of combinatorial approaches such as the co-expression of cytokines, and sets the groundwork for future developments.

## Introduction

Chimeric antigen receptor (CAR) T cell therapy was pioneered in the 1990s, and its clinical success in treating refractory hematological malignancies established it as a promising cancer immunotherapy.^1^^,^^2^^,^^3^ In addition to T cells, alternative immune cells such as natural killer (NK) cells, invariant NK T cells, γδ T cells, and macrophages have been explored for CAR cell therapy. These alternative cells offer various advantages, including innate effector functions and limited graft-versus-host reactivity, potentially enabling off-the-shelf therapies.^4^ CAR NK cells and CAR macrophages are in the focus of future developments, but previous studies with these immune effector cells mainly used immunodeficient and heterotopic mouse models, restricting our understanding of their effectiveness in the context of fully functional immune systems and orthotopic solid tumors. Additionally, a systematic comparison of distinct CAR immune effector cells is lacking.

Here, we use glioblastoma as a blueprint for a challenging solid tumor to cross-compare mouse and human CAR T cells, CAR NK cells, and CAR macrophages. Glioblastoma, the most common and aggressive primary brain tumor in adults, lacks a curative treatment and remains one of the most difficult tumors to treat.^5^^,^^6^ This underscores the need for more effective and innovative treatment strategies like CAR immune effector cell therapy. Early-phase clinical translation of CAR T cells against glioblastoma targeting single antigens such as epidermal growth factor receptor variant III (EGFRvIII), human epidermal growth factor receptor 2 (HER2), or interleukin (IL)-13 receptor alpha 2 (IL13Rα2) has shown a favorable safety profile but only modest therapeutic activity.^7^^,^^8^^,^^9^^,^^10^ Challenges include heterogeneous target antigen expression and the immunosuppressive microenvironment in glioblastoma.^11^ CAR NK cell therapies are still in the early stages of development, and preclinical studies in orthotopic immunocompetent syngeneic settings, as well as studies with primary NK cells, are limited .^12^^,^^13^ Most efforts have concentrated on CAR NK cells generated from the cell line NK-92, which needs to be pre-irradiated before *in vivo* administration. Studies on CAR macrophages against glioblastoma are rare and still limited to the preclinical stage.^14^

A cross-comparison of different CAR immune effector cells comes with several challenges that can bias the outcome of the study. These are (1) the phenotype and functional state of the immune cell that is greatly affected by the tissue origin and method of expansion/differentiation, (2) the choice of a CAR that depending on its design can elicit a different degree and quality of response in the immune effector cell after antigen binding, and (3) the tumor model, which impacts the functionality of each CAR immune effector cell.

We recently developed protocols for generating primary mouse T cells, NK cells, and macrophages under standardized conditions and extensively characterized their phenotype at different stages of differentiation to allow consistency throughout our studies.^15^^,^^16^^,^^17^ In previous studies, we extensively characterized a natural killer group 2 member D (NKG2D) CAR and its cognate ligands in the context of glioblastoma.^15^^,^^16^^,^^17^ This CAR leverages the broad binding properties of the NKG2D receptor to bind multiple cancer-associated antigens highly expressed in glioblastoma. It also exploits the naturally occurring DNAX activation proteins known to be expressed in T cells, NK cells, and macrophages as co-stimulatory domains.^18^^,^^19^ Downstream activation upon antigen binding via its conjugated CD3ζ domain together with co-stimulation via DNAX activation proteins functionally qualifies the NKG2D CAR as a second-generation CAR.^20^ The utilization of NKG2D-based CARs for CAR T cell and CAR NK cell design was successfully proven in other studies, and CD3ζ downstream signaling was described to direct anti-tumor activity in macrophages making it suitable for a cross-comparison of CAR T cells, CAR NK cells, and CAR macrophages.^21^^,^^22^^,^^23^ Together with the fact that CD3ζ downstream signaling alone was sufficient to direct anti-tumor activity in macrophages,^24^ we therefore identified the NKG2D CAR suitable for a cross-comparison of CAR immune effector cells against glioblastoma. Building on this, we here provide a cross-comparison of mouse and human NKG2D-CAR-expressing T cells, NK cells, and macrophages *in vitro* and in immunocompetent orthotopic syngeneic settings *in vivo*. Furthermore, we have extended this comparison to the functional improvement of CAR immune cells with the co-expression of pro-inflammatory cytokines, which has proven to be a safe and promising strategy in changing the brain tumor microenvironment from a cold into an immunologically hot state.

## Results

### Murine CAR T cells, CAR NK cells, and CAR macrophages can be efficiently generated using mRNA transfection and show different anti-tumor activity *in vitro*

**Figure 1:**
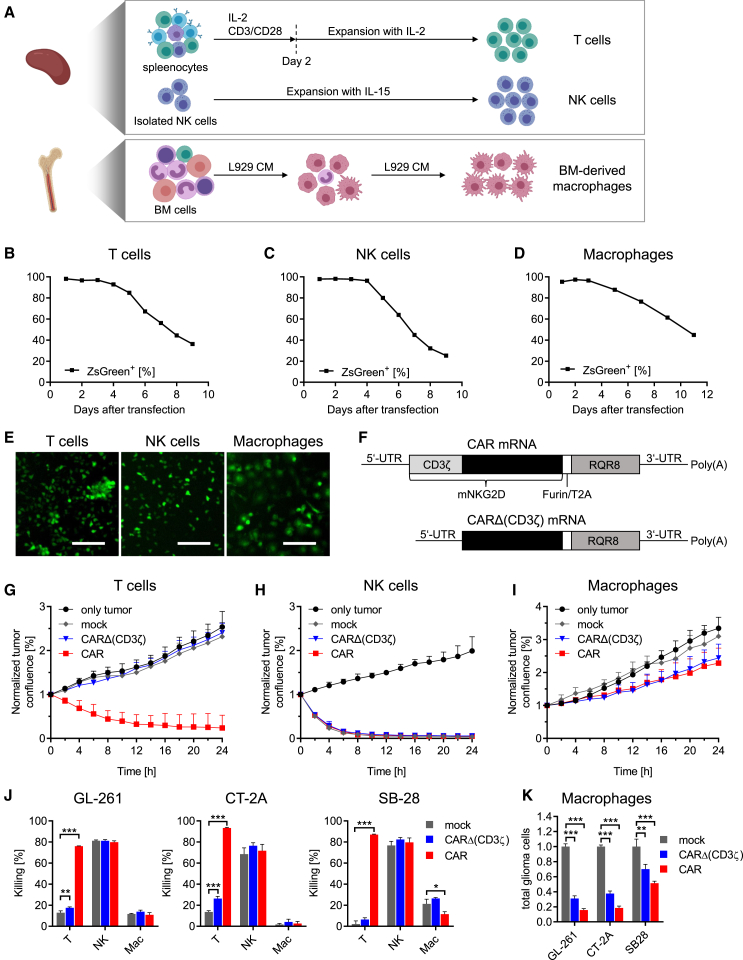
Manufacturing of mRNA-based mouse CAR immune effector cells and functional assessment thereof (A) Schematic overview for rapid and high number expansion protocols of mouse immune cells. Spleen-derived T cells are activated using αCD3 and αCD28 antibodies and expanded with IL-2. Spleen-derived NK cells are isolated using negative selection and expanded with high doses of IL-15. Macrophages are expanded from bone marrow (BM) cells using conditioned medium from L929 cells. (B–D) Mouse T cells (B), NK cells (C), or macrophages (D) were transfected with ZsGreen mRNA, and fluorescence was analyzed using flow cytometry in the following days. Quantifications for ZsGreen^+^ cells are shown. (E) Representative microcopy images of ZsGreen mRNA-transfected mouse T cells, NK cells, and macrophages one day post-transfection. Scale bars, 100 μm. (F) Schematic representation of CAR mRNA and CARΔ(CD3ζ) mRNA lacking the intracellular CD3ζ domain. Both mRNAs co-express RQR8 and were generated using *in vitro* transcription with optimized 3′-UTRs, 5′-UTRs, and pseudouridine (Ψ). (G–I) Quantifications of tdTomato^+^ GL-261 glioma cell confluence after co-cultures with mouse (G) T cells, (H) NK cells, or (I) macrophages that were mock transfected or transfected with mRNA coding for CAR or CARΔ(CD3ζ) and seeded at an E:T ratio of 2:1. Data are represented as mean + SD based on *n* = 3 FOVs. (J and K) Flow cytometry quantifications of GL-261, CT-2A, or SB-28 glioma cells that were co-cultured at an E:T ratio of 8:1 for 24 h with mouse immune cells that were mock transfected or transfected with mRNA coding for CAR or CARΔ(CD3ζ). (J) Glioma cell lysis and (K) total remaining glioma cells after co-culture are shown (mean + SD of *n* = 3, one-way ANOVA with ∗*p* < 0.05; ∗∗*p* < 0.01; ∗∗∗*p* < 0.001). FOV, field of view; E:T ratio, effector to target cell ratio; MFI, mean fluorescence intensity.

To enable a functional comparison of murine NKG2D CAR immune effector cell in syngeneic orthotopic immunocompetent settings, it was crucial to generate a sufficient number of mouse immune cells and to identify a system that allows comparable CAR expression in each cell type. For the expansion of the primary immune cell subsets, we used recently established protocols for mouse T cell, NK cell, and bone marrow-derived macrophage expansion yielding enough cells for adoptive cell transfers *in vivo* (Figure 1A).^17^ Subsequently, we tested different strategies for NKG2D CAR transgene delivery and expression. We designed mRNA, a retroviral vector, and a sleeping beauty (SB) system expressing the NKG2D CAR and the reporter protein RQR8 separated by a Furin/T2A cleavage site. RQR8 was co-expressed to quantify transfection and transduction efficiency in naturally NKG2D-positive T and NK cells. Only electroporation with mRNA demonstrated highly efficient and comparable transfection efficiencies across all cell types (Figure S1A) without hindering cell proliferation (Figures S1B–S1D). Even lentiviral transduction did not improve NK cell transfection efficiency (Figure S1E). Therefore, mRNA transfection proved to be the best system for the cross-comparison of the different CAR effector cells. To determine the kinetics of mRNA expression, we generated mRNA encoding the fluorescent protein ZsGreen. Flow cytometry revealed that electroporation with ZsGreen mRNA achieved a transfection efficiency of almost 100% for all immune cell types and ZsGreen could be detected in more than 80% of the cells for up to 5 days (Figures 1B–1D). The expression of ZsGreen was confirmed using microscopy and started 2 h after transfection until reaching a plateau after 7–12 h depending on the cell type (Figures 1E and S1F–S1H). In addition to mRNA coding for NKG2D-Furin/T2A-RQR8 (CAR), we designed a functional control encoding solely the NKG2D tumor binding domain without the intracellular CD3ζ domain (CARΔ(CD3ζ)) (Figure 1F). To assess the anti-tumor potential of the different CAR immune effector cells *in vitro*, we co-transfected them with mRNA coding for ZsGreen and mRNA coding for CAR or control CARΔ(CD3ζ), co-cultured them with tdTomato-expressing GL-261 cells, and quantified tumor confluence over time. Only CAR T cells reduced tumor cell confluence whereas CARΔ(CD3ζ) T cells or mock-transfected T cells showed no effect compared to tumor cells only (Figure 1G and Video S1. Pre-seeded GL-261 + mock T cells, related to Figure 1, Video S2. Pre-seeded GL-261 + CARΔ(CD3ζ) T cells, related to Figure 1, Video S3. Pre-seeded GL-261 + CAR T cells, related to Figure 1, Video S4. Pre-seeded GL-261 only, related to Figure 1). NK cell-mediated killing was independent of CAR expression and displayed fast kinetics with almost complete eradication of glioma cells 8 h after co-culture (Figure 1H and Video S4. Pre-seeded GL-261 only, related to Figure 1, Video S5. Pre-seeded GL-261 + mock NK cells, related to Figure 1, Video S6. Pre-seeded GL-261 + CARΔ(CD3ζ) NK cells, related to Figure 1, Video S7. Pre-seeded GL-261 + CAR NK cells, related to Figure 1). The lack of improved NK cell killing after CAR expression made us wonder if this is due to the CAR design or due to overactivation of NK cells by *in vitro* culture conditions. We investigated this by transfecting NK cells with a second-generation CD28^−^CD3ζ CAR targeting CD19 on lymphoma cells. CD19 CAR expression showed modest improvement in target cell killing while T cell killing was drastically increased (Figures S2A and S2B). This suggests that other CAR designs might increase NK cell killing of glioma cells. However, under immunosuppressive conditions that downregulated NKG2D surface expression and anti-tumor activity of mock-transfected NK cells, NKG2D CAR expression retained NK cell functionality, proving a functional benefit (Figures S2C–S2E). This made us continue using the NKG2D CAR in our study. Macrophages reduced tumor cell confluence over time when expressing CAR or CARΔ(CD3ζ) but did not control tumor growth (Figures 1I; Video S8. Co-seeded GL-261 + mock macrophages, related to Figure 1, Video S9. Co-seeded GL-261 + CARΔ(CD3ζ) macrophages, related to Figure 1, Video S10. Co-seeded GL-261 + CAR macrophages, related to Figure 1, Video S11. Co-seeded GL-261 only, related to Figure 1). CAR expression on macrophages favored CD86 expression, a known marker for a pro-inflammatory macrophage phenotype (Figure S2F). Flow cytometry on co-cultures with three different glioma cells lines and two brain metastasis cell lines confirmed the findings and demonstrated that macrophages reduced tumor cell numbers without tumor cell lysis (Figures 1J, 1K, and S2G–S2K). Major histocompatibility complex class I (MHC class I) molecules but not NKG2D ligand Rae-1 surface expression inversely correlated with NK cell-mediated killing of cell lines (Figure S2L).

Video S1. Pre-seeded GL-261 + mock T cells, related to Figure 1

Video S2. Pre-seeded GL-261 + CARΔ(CD3ζ) T cells, related to Figure 1

Video S3. Pre-seeded GL-261 + CAR T cells, related to Figure 1

Video S4. Pre-seeded GL-261 only, related to Figure 1

Video S5. Pre-seeded GL-261 + mock NK cells, related to Figure 1

Video S6. Pre-seeded GL-261 + CARΔ(CD3ζ) NK cells, related to Figure 1

Video S7. Pre-seeded GL-261 + CAR NK cells, related to Figure 1

Video S8. Co-seeded GL-261 + mock macrophages, related to Figure 1

Video S9. Co-seeded GL-261 + CARΔ(CD3ζ) macrophages, related to Figure 1

Video S10. Co-seeded GL-261 + CAR macrophages, related to Figure 1

Video S11. Co-seeded GL-261 only, related to Figure 1

### CAR T cells demonstrate superior accumulation in the tumor *in vivo*

**Figure 2:**
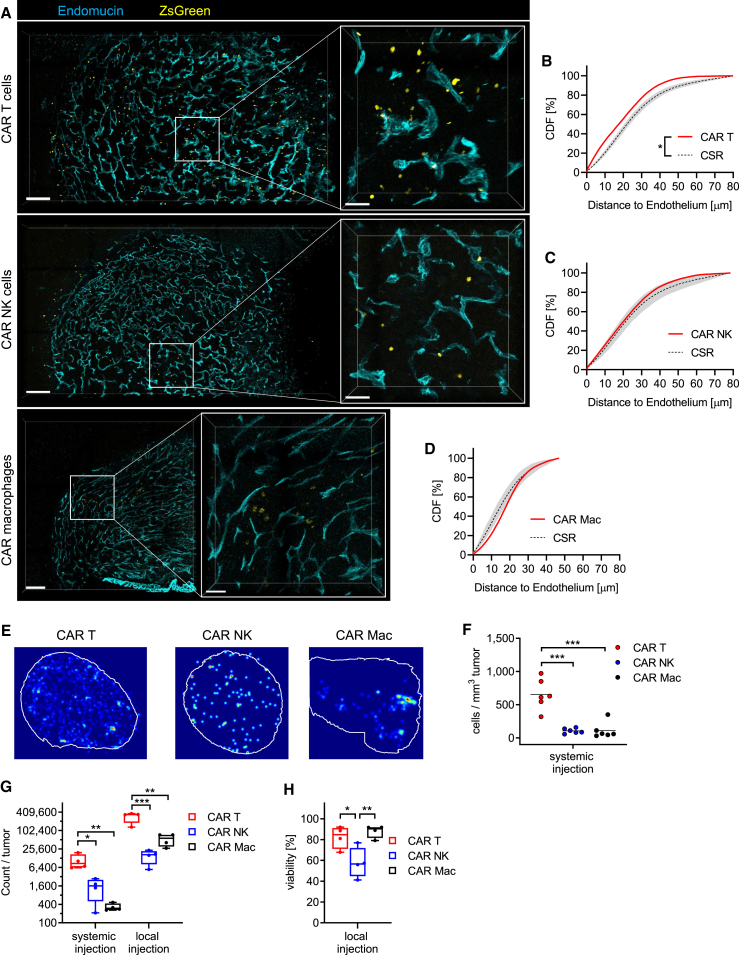
3D confocal microscopy and flow cytometry unveil the tumor-infiltrative capacity of CAR T cells, CAR NK cells, and CAR macrophages (A–F) GL-261 iRFP720 glioma-bearing C57BL/6 mice received intravenous injections of 5 × 10^6^ CAR immune effector cells co-expressing ZsGreen on day 11 after glioma cell implantation. Mice were perfused two days later, and brain sections stained for DAPI and the endothelial cell marker endomucin (Emcn). (A) Representative 3D images of tumor-bearing hemispheres with Emcn^+^ endothelium (turquoise) and ZsGreen^+^ CAR immune effector cells (yellow). Scale bars are 200 μm (left) and 50 μm (right). (B–D) CDF plots of distances to the endothelium are shown in red compared to the simulated CSR distributions in gray for CAR T cells (B), CAR NK cells (C), and CAR macrophages (D). Two-sample Kolmogorov-Smirnov test was used to analyze significance (CDF versus CSR plots: ∗*p* < 0.05). (E) Representative 2D tissue maps display the spatial distribution of CAR T cells, CAR NK cells, and CAR macrophage as single cells or clusters within whole tumor sections. (F) Quantification of tumor-infiltrating CAR T cells, CAR NK cells, and CAR macrophages in brain slices as cell numbers per tumor volume (*n* = 6, one-way ANOVA with ∗*p* < 0.05; ∗∗*p* < 0.01; ∗∗∗*p* < 0.001). (G and H) GL-261 iRFP720 glioma-bearing CD45.2^+^ C57BL/6 received either intravenous injections of 5 × 10^6^ CD45.1^+^ CAR immune effector cells or local injections of 2 × 10^6^ CD45.1^+^ CAR immune effector cells on day 11 after glioma cell implantation. Mice were perfused two days later, and tumor-bearing hemispheres dissociated. (G) Absolute cell counts per tumor and (H) the viability of CD45.1^+^CD11b^−^CD3^+^ CAR T cells, CD45.1^+^CD11b^−^CD335^+^ NK cells, and CD45.1^+^CD11b^+^ CAR macrophages were quantified using flow cytometry (boxplot with median +/− quartiles and min to max of *n* = 4, one-way ANOVA with ∗*p* < 0.05; ∗∗*p* < 0.01; ∗∗∗*p* < 0.001). CDF, cumulative distribution function; CSR, complete spatial random.

To evaluate the tumor homing properties of systemically administered CAR T cells, CAR NK cells, and CAR macrophages in an orthotopic, syngeneic solid tumor setting, we characterized the quantity and spatial distribution of intravenously administered fluorescently labeled NKG2D CAR immune effector cells in the brains of GL-261 glioma-bearing mice using *ex vivo* 3D microscopy (Figure S3A). All types of adoptively transferred CAR immune effector cells were predominantly located within the highly vascularized tumoral mass and not the surrounding brain parenchyma (Figure 2A and Video S12. 3D confocal imaging of CAR T cells within a GL-261 brain tumor, related to Figure 2, Video S13. 3D confocal imaging of CAR NK cells within a GL-261 brain tumor, related to Figure 2, Video S14. 3D confocal imaging of CAR macrophages within a GL-261 brain tumor, related to Figure 2). Detailed spatial analysis of cumulative CAR immune effector cell distributions compared to a random simulation revealed a tendency for CAR T cells, but not CAR NK cells and CAR macrophages, to reside in close proximity to vascular structures (Figures 2B–2D and S3B–S3D). The relative vasculature volume per tumor was comparable after each treatment (Figure S4A). Of note, while both CAR T and NK cells homogeneously distributed throughout all areas of the tumor, CAR macrophages accumulated in clusters and were almost absent in the outer zones of the tumor (Figures 2E and S4B). Among the different effector cells, CAR T cells were more abundant than CAR NK cells or CAR macrophages (Figure 2F). To confirm the 3D microscopy analysis, we intravenously injected CD45.1^+^ immune cells, transfected with NKG2D CAR mRNA, into CD45.2^+^ glioma-bearing mice and quantified the number of tumor-infiltrating cells using *ex vivo* flow cytometry (Figure S3A). This confirmed that CAR T cells were more abundant than CAR NK cells or CAR macrophages (Figure 2G). Because of the low cell numbers, we also investigated local intratumoral injections as an alternative administration route. This drastically increased the intratumoral numbers of all CAR immune effector cells compared to systemic injection. Two days after intratumoral administration, CAR T cells and CAR macrophages had a high viability within the tumor, whereas CAR NK cells displayed a lower viability (Figure 2H). Overall, these results suggest that CAR T cells have the best tumor homing potential upon intravenous administration, but in general the local administration route is preferred to get sufficient CAR effector cells to the tumor site.

Video S12. 3D confocal imaging of CAR T cells within a GL-261 brain tumor, related to Figure 2

Video S13. 3D confocal imaging of CAR NK cells within a GL-261 brain tumor, related to Figure 2

Video S14. 3D confocal imaging of CAR macrophages within a GL-261 brain tumor, related to Figure 2

### scRNA-seq identifies distinct immune signatures for CAR T cell, CAR NK cell, and CAR macrophage therapy in the tumor microenvironment

To characterize the effects of the different CAR effector cells on the tumor microenvironment, we administered CD45.1^+^ CAR T cells, CAR NK cells, or CAR macrophages intratumorally in GL-261 glioma-bearing mice and profiled the landscape of CD45.2^+^ tumor-infiltrating immune cells 5 days after the treatment using single-cell RNA sequencing (scRNA-seq) (Figure S3A). We assessed single-cell RNA profiles of at least *n* = 6,499 leukocytes per sample that passed the quality controls (Figure S5A). The resulting *n* = 33,345 cells were visualized using uniform manifold approximation and projection (UMAP).

**Figure 3:**
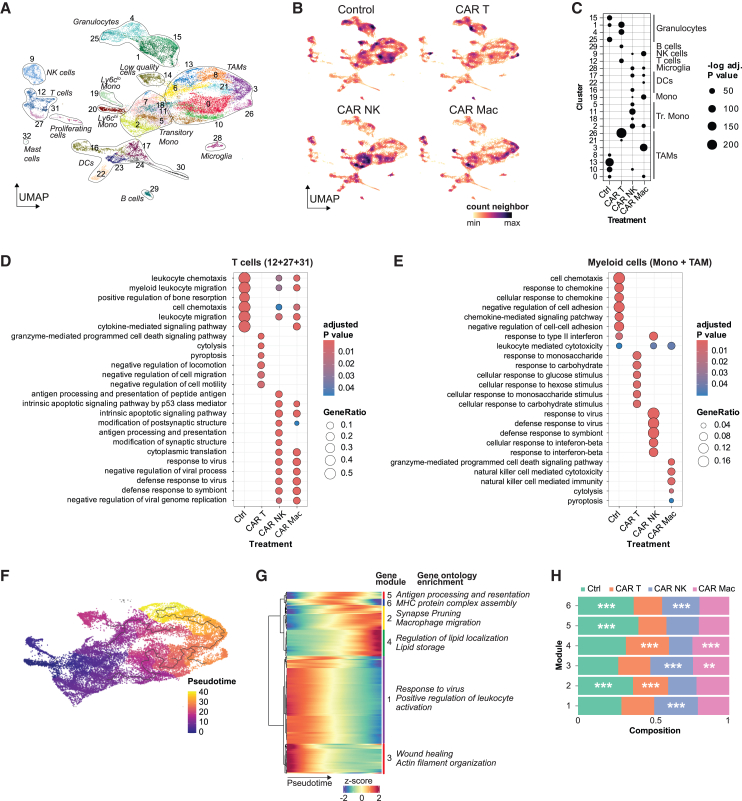
Single-cell RNA sequencing identifies CAR immune effector cell treatment-specific signatures in the tumor microenvironment of GL-261-tumor-bearing mice GL-261 glioma cells were implanted orthotopically into the brains of C57BL/6 wild-type mice. On day 7 after implantation, 2 × 10^6^ CAR T cells, CAR NK cells, or CAR macrophages were injected intratumorally, and 5 days later the tumor was isolated and dissociated. CD45^+^ immune cells were sorted with fluorescence-activated cell sorting and subjected to scRNA-seq. (A) UMAP representation of 36,142 CD45^+^ cells that passed quality control. (B) Visualization of the local abundance of the different conditions. The color coding represents the presence of cells of the same condition across the different neighborhoods within the UMAP latent space. (C) Differential enrichment testing of cells from the respective conditions based on hypergeometric testing with Benjamini-Hochberg adjustment for multiple testing. (D and E) Treatment-dependent enrichment of gene ontology terms across T cells and myeloid cells. The dot size indicates the gene ratios of differentially expressed genes found in the present dataset versus genes contained in the respective gene ontology term. The color coding represents the *p* value of the underlying enrichment test. (F) UMAP of the myeloid cell clusters color-coded for the pseudotime calculated using monocle3. The line represents a path of stepwise gene expression changes. (G) Visualization of the stepwise gene expression along the pseudotime trajectory with the representative gene ontology terms enriched in the respective gene expression modules along the trajectory. (H) Differential enrichment testing of the different treatment conditions across the gene expression modules along the pseudotime trajectory.

The landscape of tumor-infiltrating immune cells in glioblastoma comprised different myeloid cell clusters including monocytes, a large cluster of tumor-associated macrophages (TAMs), and microglia (Figure 3A; Figure S5B). Furthermore, we detected granulocytes, dendritic cells, NK cells, T cells, and B cells. The different CAR immune effector cells led to an individual polarization of the tumor immune microenvironment. CAR T cell treatment was markedly associated with a higher presence of TAMs and granulocytes. CAR NK cell and CAR macrophage treatments were prominently associated with a higher abundance of transitory monocytes or TAMs and NK cells, respectively (Figures 3B and 3C).

Gene ontology enrichment analysis of the T cell cluster and the myeloid cell clusters comprising monocytes and TAMs further corroborated these distinct effects (Figures 3D and 3E). CAR T cell treatment caused a shift of the T cell clusters toward a cytotoxic state, whereas the effect on the myeloid compartment appeared mostly metabolically and was characterized by changes in glycolytic transcriptional programs. Treatment with CAR NK cells and CAR macrophages induced ontology terms associated with anti-viral immune defense (Figure 3D). A shift toward an anti-viral immune response was also seen in myeloid cells after CAR NK cell and CAR macrophage treatment (Figure 3E). However, for CAR macrophages, it appeared not cell autonomous and driven via NK cells.

Next, we further dissected the different effects of CAR immune effector cell treatment on the myeloid compartment, representing the largest immune cell subset within the glioma tumor microenvironment. For this, we conducted pseudotime analysis with the classical Ly6c^hi^ monocyte cluster as the earliest time point (Figure 3F). On the identified transcriptional continuum, transitory monocytes were followed by TAMs with increasing expression of microglia-associated transcriptional programs (Figure S4B). Analysis of significantly enriched genes within the six emerging gene modules showed that early “pseudo”-time points were associated with wound healing and anti-viral response terms. Intermediate time points were characterized by antigen processing-associated genes and transitioning to macrophage migration; late time points displayed lipid metabolism states (Figure 3G). Abundance analysis of the respective stages showed enrichment of control groups at intermediate- and late-stage-associated gene expression modules (Figure 3H). Likewise, CAR T cell treatment was associated with intermediate- and late-stage-associated modules 2 and 4, while CAR NK cell treatment was significantly associated with the early- and intermediate-stage modules 1, 3, and 6. CAR macrophage treatment showed an ambiguous profile with enrichment in the early-stage module 3 (along with CAR NK cells) and the late-stage module 4 (along with CAR T cells). In summary, CAR T cell treatment was associated with a metabolic rewiring of myeloid cells and the emergence of late-stage TAMs while CAR NK cell treatment increased the abundance of transitory monocytes and anti-viral responses across T cells and myeloid cells. CAR macrophage treatment showed features of both.

### A limited survival benefit of CAR immune effector cell treatment can be turned into a curative treatment by co-expression of pro-inflammatory cytokines

**Figure 4:**
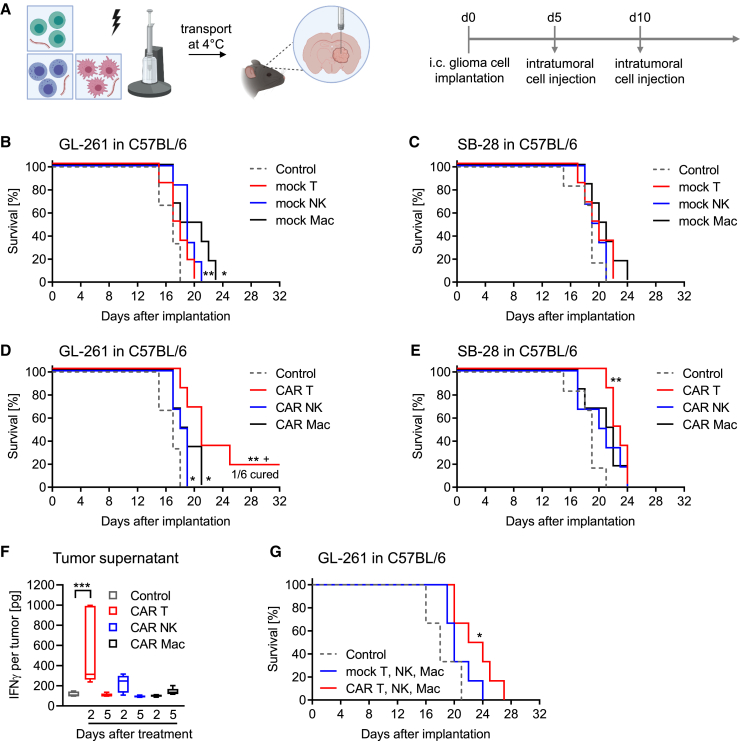
CAR immune effector cells show limited efficacy in immunocompetent syngeneic glioma mouse models (A) Schematic overview of CAR immune effector cell generation using mRNA electroporation; treatment of orthotopic, immunocompetent glioma mouse models; and the treatment schedule. (B–G) GL-261 or SB-28 glioma cells were implanted orthotopically in C57BL/6 wild-type mice. Mice received intratumoral treatment with either 2 × 10^6^ mock-transfected immune cells (B and C) or 2 × 10^6^ CAR immune effector cells (D–F) or an equal mix of 2 × 10^6^ CAR immune effector cells (G). For survival, mice were treated on day 5 and day 10 after tumor implantation as shown in the scheme. Survival data of *n* = 6 mice per treatment group are presented as Kaplan-Meier plots. *p* values were calculated with log rank test (treatment versus control: ∗*p* < 0.05; ∗∗*p* < 0.01, ∗∗∗*p* < 0.001). (F) Mice received intratumoral CAR immune effector cell treatment 7 days after tumor implantation, and tumor supernatants were collected 2 or 5 days later to quantify IFNγ concentration using ELISA (boxplot with median +/− quartiles and min to max of *n* = 5, one-way ANOVA with ∗*p* < 0.05; ∗∗*p* < 0.01, ∗∗∗*p* < 0.001.).

**Figure 5:**
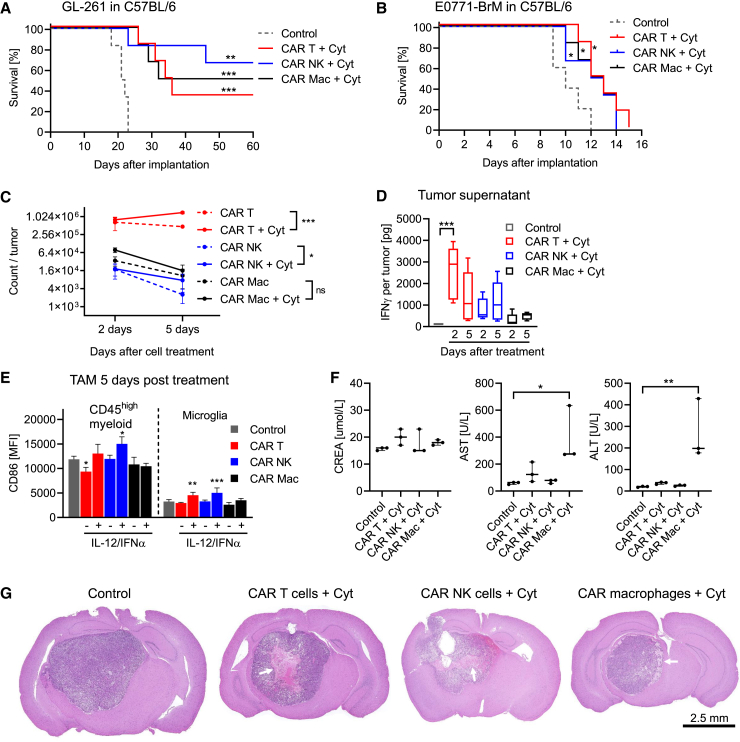
Limited efficacy of CAR immune effector cells can be overcome by co-expression of inflammatory cytokines (A and B) GL-261 glioma or E0771-BrM breast cancer cells were implanted orthotopically in C57BL/6 wild-type mice. Mice received intratumoral treatment with 2 × 10^6^ multifunctional CAR immune effector cells on day 5 and day 10 after tumor implantation. Survival data of *n* = 6 mice per treatment group are presented as Kaplan-Meier plots. *p* values were calculated with log rank test (treatment versus control: ∗*p* < 0.05; ∗∗*p* < 0.01, ∗∗∗*p* < 0.001). (C–E) Mice received intratumoral CAR immune effector cell treatment or multifunctional CAR immune effector cell treatment 7 days after tumor implantation, and tumors were collected 2 or 5 days later. (C) Quantification of tumor-infiltrating adoptively transferred CAR immune effector cells 2 or 5 days after injection (mean ± SD of *n* = 5, unpaired t test with ∗*p* < 0.05; ∗∗*p* < 0.01, ∗∗∗*p* < 0.001). (D) Quantification of IFNγ concentration in tumor supernatant 2 or 5 days after CAR immune effector cell injection using ELISA (boxplot with median +/− quartiles and min to max of *n* = 5, one-way ANOVA with ∗*p* < 0.05; ∗∗*p* < 0.01, ∗∗∗*p* < 0.001). (E) CD86 surface expression on tumor-infiltrating CD45^high^ myeloid cells and microglia 5 days after CAR immune effector cell injection as quantified by flow cytometry (mean + SD of *n* = 5, one-way ANOVA with ∗*p* < 0.05; ∗∗*p* < 0.01, ∗∗∗*p* < 0.001. (F) Blood of mice from (A) was collected 12 days after tumor cell implantation. Blood of two mice each was pooled, and clinical parameters (CREA, AST, ALT) were analyzed (boxplot with median +/− quartiles and min to max of *n* = 3, one-way ANOVA with ∗*p* < 0.05; ∗∗*p* < 0.01). (G) Representative pictures of H&E-stained coronal sections of tumor-bearing mouse brains from (A) at 1.25× magnification. The white arrow indicates areas of necrosis. CREA, creatinine; AST, aspartate aminotransferase; ALT, alanine transaminase.

Next, we compared the therapeutic potential of the different CAR immune effector cells *in vivo*. For this, we used fully immunocompetent syngeneic glioma mouse models and administered CAR T cells, CAR NK cells, or CAR macrophages intratumorally (Figure 4A). Treatment with mock-transfected T cells, NK cells, or macrophages had a limited effect on overall survival without long-term surviving mice (Figures 4B and 4C). Among all CAR immune effector cells, CAR T cells performed best. However, the overall anti-tumor activity was still limited, with only one long-term surviving GL-261 tumor-bearing mouse and only improved median survival without long-term survival in SB-28 tumor-bearing mice (Figures 4D and 4E). CAR T cell treatment was also accompanied with the strongest interferon (IFN)γ release within the tumor two days after treatment whereas IFNγ concentrations within the plasma remained below detection limit (Figures 4F and S6A). The marginal survival effect was not linked to a decline in CAR cell viability during the treatment administration process. CAR immune effector cells that persisted after surgery maintained high viability and retained *in vitro* anti-tumor activity (Figures S6B–S6D). The individual advantages of each CAR immune effector cell prompted an exploration into whether their combined administration could elicit synergistic effects *in vivo*. However, this was not the case, as a combined mixture of CAR T cells, CAR NK cells, and CAR macrophages improved the median overall survival but did not lead to higher numbers of long-term surviving mice (Figure 4G). To overcome the immunosuppressive tumor microenvironment, we recently explored and demonstrated that multifunctional CAR T cells, additionally transfected with mRNA encoding for the cytokines IL-12 and IFNα2, were able to cure glioma-bearing mice.^16^ Consequently, we investigated whether also the co-transfection of NK cells and macrophages with CAR-, IL-12-, and IFNα2-encoding mRNAs could similarly improve the survival of glioma-bearing mice. Indeed, treatment with each multifunctional CAR immune effector cells improved overall survival, with multifunctional CAR NK cells demonstrating the best performance and curing 4 out of 6 glioma-bearing mice (Figure 5A). Even treatment with T cells and NK cells, transfected with mRNA coding only for the cytokines without the CAR, improved overall survival of glioma-bearing mice (Figure S6E). A survival benefit from cytokine co-expression in CAR immune effector cells was also seen in mice bearing the aggressive breast cancer metastasis cell line E0771-BrM (Figures 5B and S6F). In glioma-bearing mice, cytokine co-expression was shown to increase CAR T cell and CAR NK cell numbers five days after injection. This correlated with prolonged intratumoral IFNγ release and CD86 upregulation on tumor-associated myeloid cells (Figures 5C, 5D, and S6G). To the same extent, CD86 expression on CAR macrophages was increased by cytokine co-expression and increased numbers of CD8α T cells recruited to the tumor (Figures S6H and S6I).

Overall, treatment was tolerated well and bodyweights, serving as indirect indicators for toxicity, remained stable throughout multifunctional CAR and cytokine-expressing immune cell therapy (Figures S6J and S6K). Blood value assessment revealed unchanged creatinine values but elevated aspartate aminotransferase (AST), alanine transaminase (ALT), and IFNγ values for multifunctional CAR macrophages (Figures 5F, S6L, and S6M). However, histologic evaluation showed no significant morphological changes related to multifunctional CAR cell administration within the livers and the spleens of euthanized mice (Figures S7A and S7B). Brain neoplasms showed regions of coagulative and lytic necrosis after multifunctional CAR and cytokine-expressing immune cell treatment (Figures 5G and S7C); however, no obvious changes other than inflammatory and necrotizing changes associated with the neoplasm were observed in the brain of euthanized tumor-bearing mice. These data confirm a safe administration of multifunctional CAR immune effector cells and their superior anti-tumor activity compared to immune cells expressing only the CAR *in vivo*.

### Only CAR lymphocytes demonstrate activity against human glioblastoma *in vitro* and *ex vivo*

**Figure 6:**
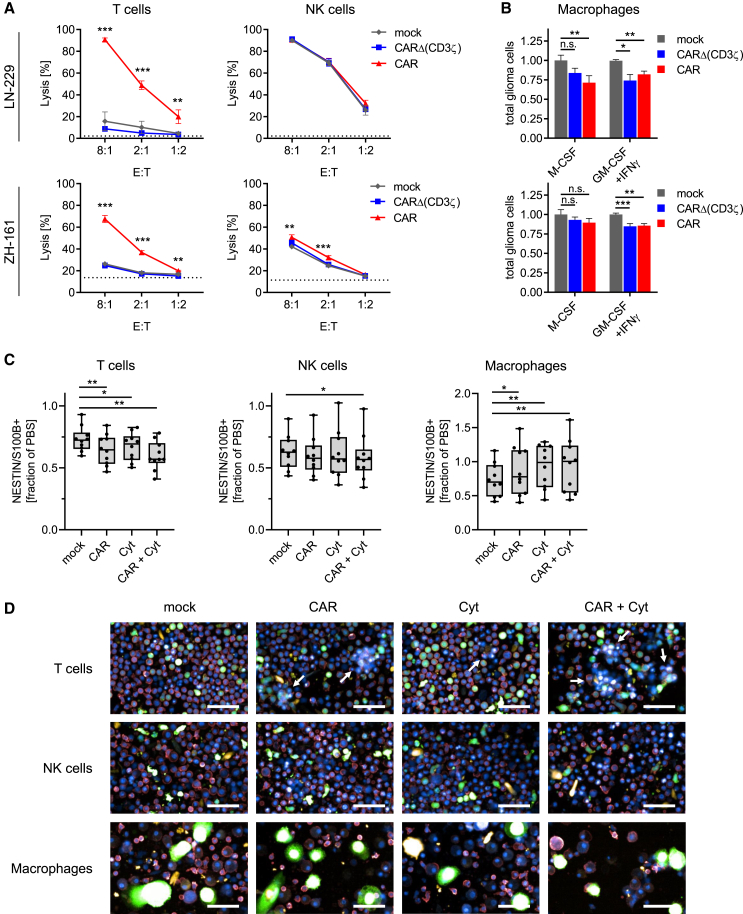
Human CAR and multifunctional CAR lymphocytes kill glioma cell lines and glioblastoma cells from patient samples with an intact microenvironment (A) Human LN-229 and ZH-161 glioma cells were co-cultured with human T cells or NK cells that were mock transfected or transfected with mRNA coding for CAR or CARΔ(CD3ζ) at different E:T ratios for 24 h. Glioma cell lysis was assessed using flow cytometry (mean ± SD of *n* = 3, one-way ANOVA with ∗*p* < 0.05; ∗∗*p* < 0.01; ∗∗∗*p* < 0.001). (B) Same setup as in (A) but with human macrophages that were polarized using M-CSF or GM-CSF/IFNγ and co-cultured at an E:T ratio of 8:1. Total remaining glioma cells after co-culture are shown (mean + SD of *n* = 3, one-way ANOVA with ∗*p* < 0.05; ∗∗*p* < 0.01; ∗∗∗*p* < 0.001). (C and D) Human immune cells transfected with ZsGreen mRNA and mRNA coding for CAR or cytokines or both were *ex vivo* co-cultured with surgically derived patient glioblastoma samples, and images of co-cultures were quantified using pharmacoscopy. (C) Remaining fractions of glioblastoma cells from *n* = 10 glioblastoma patient samples are represented as boxplot with median +/− quartiles and min to max. Significances were quantified by comparing co-cultures with mock-transfected immune cells and co-cultures with mRNA-transfected immune cells using paired nonparametric t test (∗*p* < 0.05; ∗∗*p* < 0.01). (D) Representative immunofluorescence images of co-cultures are shown with CD45^+^ immune cells in red, tumor cells in orange, and CAR immune cells in green. White arrows denote immune cell clusters. Scale bar, 40 μm.

Finally, we aimed to determine if the findings observed with murine CAR effector cells could be translated to human CAR immune effector cells. For this, we established protocols for the expansion of primary human T cells, NK cells, and macrophages (Figures S8A–S8F). After confirming high mRNA transfection efficacies of >94% for all cell types (Figure S8G), we conducted 24 h killing assays using the adherent human glioma cell line LN-229 and the sphere-forming glioma-initiating cell line ZH-161. Similar to their mouse counterparts, human CAR T cells but not control CARΔ(CD3ζ)-expressing T cells efficiently lysed both cell lines. In contrast, NK cell killing was only improved by CAR expression under immunosuppressive conditions that downregulate NKG2D surface expression like the murine counterpart (Figure 6A; Figures S8H–S8J). Contrary to mouse cells, human NK cell-mediated killing did not correlate with surface expression of MHC class I molecules on tumor cells but correlated with NKG2D ligands MHC class I polypeptide-related sequence A/B (MICA/B) (Figure S8K). Macrophages were differentiated from CD14^+^ monocytes using macrophage colony-stimulating factor (M-CSF) or polarized with the pro-inflammatory cytokines granulocyte-macrophage colony-stimulating factor (GM-CSF) and IFNγ. Both M-CSF and GM-CSF/IFNγ differentiated CAR macrophages and led to a glioma cell reduction of up to 30%, whereas control CARΔ(CD3ζ)-expressing macrophages reduced glioma cell numbers to a lesser extent (Figure 6B). Next, we co-transfected human immune cells with mRNA encoding ZsGreen and mRNA encoding the human NKG2D CAR, human cytokines (IL-12 and IFNα2), or both, CAR and cytokines, and co-cultured them with glioblastoma patient samples *ex vivo*. After 24 h, we analyzed the number of glioblastoma cells using pharmacoscopy, an image-based single-cell platform. Overall, tumor cell fractions were notably reduced if co-cultured with human lymphocytes compared to PBS control (Figure 6C). Additionally, we observed improved anti-glioblastoma activity for CAR T cells and CAR T cells co-expressing cytokines as shown by reduced tumor cell fractions and the presence of T cell clustering for T cells (Figures 6C and 6D). Furthermore, co-expression of the CAR and cytokines significantly improved NK cell killing of tumor cells. These results demonstrate a superiority of human CAR lymphocytes over human CAR macrophages in glioblastoma cell killing and support an anti-tumor benefit if CAR lymphocytes are transfected to co-express IL-12 and IFNα2.

## Discussion

Understanding advantages and limitations of different CAR immune effector cells is crucial for the development of the next generation of adoptive cell therapy strategies. This includes logistical features as well as therapeutic aspects like safety and mode of action. CAR NK cells and CAR macrophages are particularly known for their appealing safety profile with reduced risk for cytokine release syndrome as compared to CAR T cells.^25^^,^^26^ Furthermore, they do not induce graft-versus-host-disease enabling potential use as off-the-shelf allogenic cell therapy. In this study, we focused on therapeutical aspects and characterized the tumor homing, anti-tumor potential, tumor microenvironment-shaping properties, and *in vivo* toxicities of CAR T cells, CAR NK cells, and CAR macrophages in the context of a challenging solid tumor, glioblastoma. *In vitro*, T cell-mediated killing was CAR dependent, whereas NK cells displayed strong cytotoxicity independent of the CAR, underscoring their spontaneous cytolytic activity and potential to eliminate even antigen-negative tumor cells.^27^ Natural effector functions of macrophages are the modulation of immune responses by secretion of soluble mediators and phagocytosis. Accordingly, CAR macrophages displayed limited tumor cell lysis *in vitro* but reduced the number of cancer cells, consistent with prior research.^24^ For *in vivo* studies, we employed orthotopic syngeneic mouse glioma models, allowing us to examine toxicities and microenvironmental effects of CAR immune effector cells in fully immunocompetent hosts. Administrations of all CAR immune effector cells were well tolerated, with no signs of direct or indirect toxicity. Among the different CAR immune effector cells, CAR T cells exhibited superior tumor homing compared to CAR NK cells and CAR macrophages upon intravenous administration. However, consistent with other studies, overall accumulation in the tumor upon systemic administration was inefficient, emphasizing the importance of efforts to facilitate tumor homing, such as overexpression of chemokines or matrix-digesting enzymes, which have already been investigated for CAR T cells. Our data provide a rationale to investigate these strategies also for CAR NK cells and CAR macrophages.^28^ To overcome this limitation, we focused on local intratumoral administrations as an alternative route for comparing anti-tumor properties and microenvironmental effects *in vivo*. Different CAR immune effector cells had distinct pharmacodynamic effects on the tumor microenvironment in fully immunocompetent hosts. CAR T cells were accompanied by an influx of granulocytes and increased intratumoral IFNγ levels, whereas CAR NK cells promoted tumor infiltration of transitory monocytes with gene enrichment for anti-viral pathways, providing initial insights into the crosstalk of different CAR immune effector cells with tumor cells and bystander immune cells. This information might be leveraged for future approaches supporting bystander cells specific to the CAR immune effector cell type. Despite these effects on the tumor microenvironment, the overall survival benefit was limited for all effector cells, emphasizing the need for optimized and more potent CAR immune effector cell approaches. We and others have previously demonstrated that pro-inflammatory cytokines can transform the immunologically cold microenvironment into a hot one^29^^,^^30^^,^^31^ and that the co-expression of the pro-inflammatory cytokines IL-12 and IFNα2 enhances the anti-tumor efficacy of CAR T cells and bystander immune cells within a tumor.^16^ Here, we extended this strategy to CAR NK cells and CAR macrophages and achieved promising responses with a large proportion of long-term surviving mice. Cytokine co-expression correlated with increased CAR T cell and CAR NK cell persistence and prolonged intratumoral IFNγ release. Furthermore, it led to increased myeloid cell activation and CD8α T cell infiltration in the tumor. For this study, we chose mRNA as the delivery method for the transgenes. This is a potential limitation because it only allows transient expression and requires multiple dosing. On the other hand, it has been demonstrated for T cells that persistence is associated with T cell hypofunction and that multiple-dosing strategies might prevent T cell hypofunction resulting from persistence in the immunosuppressive environment, which supports the mRNA-based approach.^28^

Other limitations of this study include the origin of immune cells and the CAR construct. To have sufficient mouse immune cell numbers for syngeneic studies, we were restricted to expand NK cells with high doses of IL-15 and to differentiate macrophages from bone marrow progenitors. This might lead to deviating results as would have been obtained from other immune cell sources like peripheral blood, umbilical cord blood, and hematopoietic progenitors as currently investigated in the clinic. Further, different culture conditions like co-culture with feeder cells or supplementation with pre-activating cytokines could impact the outcome after CAR NK cell and CAR macrophage therapy in this study. Another confounding variable impacting the quality of results is the CAR design. Our study intentionally aimed at minimizing CAR immune cell-specific functional improvements to reduce the number of variables that complicate the interpretation of results of the cross-comparison. Therefore, we used the same NKG2D CAR design for all CAR immune effector cells. With a DNAX activating protein binding site and CD3ζ signaling domain, the NKG2D CAR is a functionally second-generation CAR, which is the most commonly used CAR design for current CAR T, CAR NK, and CAR macrophage approaches. However, other intracellular signaling domains have been described and might be more advantageous to promote the effector functions of CAR NK cells and CAR macrophages.^24^^,^^32^^,^^33^ For example, substituting the CAR DNAX activating protein binding domain with a 2B4 co-stimulatory domain could improve the anti-glioma activity of CAR NK cells *in vitro* and *in vivo.*^33^

Our data encourage further focus on elaborating cell-intrinsic limitations of CAR immune effector cell therapies and implementing these findings in better CAR immune effector cell designs or combination therapies. Future approaches can be combination therapies with immune checkpoint inhibitors or treatment combinations that additionally stimulate the corresponding bystander cells associated with a certain CAR effector cell. Furthermore, we demonstrate that multifunctional CAR immune effector cells hold potential as an effective immunotherapy against glioblastoma.

### Limitations of the study

For this study, mRNA was used that allowed immune cell transfection with high efficiency; however, protein expression is only transient. Different outcomes might be observed if CAR immune cells are generated with stable transfection methods. Cell expansion protocols were optimized for fast and high-yield immune cell expansion. Other tissue origin and cell stimulation techniques can lead to different results. This study was based on an NKG2D CAR with an intracellular CD3ζ domain. This CAR might not be the most optimal for each immune cell used in the study, and other CAR designs should be considered for follow-up studies trying to optimize CAR immune cells. Performing intracellular signaling studies can be helpful in identifying optimized CARs for each immune cell.

## Resource availability

### Lead contact

Further information and requests for resources and reagents should be directed to and will be fulfilled by the lead contact, Tobias Weiss (tobias.weiss@usz.ch).

### Materials availability

This study did not generate new unique reagents.

### Data and code availability

• scRNA-seq data have been deposited at GEO at GEO: GSE283049 and are publicly available as of the date of publication.
• All original code has been deposited at Zenodo and is publicly available at DOI: https://doi.org/10.5281/zenodo.14230743 as of the date of publication.
• Any additional information required to reanalyze the data reported in this paper is available from the lead contact upon request.

## Acknowledgments

We thank Obinna Chijioke for kindly providing the K562-mbIL21 feeder cells and Chiara Magnani for providing the pT4 sleeping beauty plasmid backbone. We also thank Charles L. Sentman for providing the NKG2D CAR. Flow cytometry was performed with equipment of the flow cytometry facility, University of Zurich. This study was supported by the Highly Specialized Medicine (HSM) Program of the Canton of Zurich and the Clinical Research Priority Program (CRPP) of the University of Zurich for the CRPP ImmunoCure (to M.W.), the Comprehensive Cancer Center Zurich (CCCZ) Lighthouse Project IMMUNO-CAR ZURICH (ZURICAR) (to T.W.), the Promedica Foundation (to T.W.), the Baasch-Medicus Foundation (to T.W.), the 10.13039/501100013850Helmut Horten Foundation (to T.W.), and Sophien Foundation (to T.W. and M.W.). Furthermore, T.W. received support from the Swiss Cancer Research League, grant KFS-4533-08-2018-R.

## Author contributions

T.L. and T.W. designed and conducted *in vitro* and *in vivo* experiments, analyzed the data, and wrote the manuscript. T.L., T.W., and S.P. developed and optimized the mRNA-based immune cell transfection methodology. R.S. and N.B. conducted, analyzed, and interpreted scRNA-seq. M.B. conducted *in vitro* and *in vivo* experiments. S.F. and C.N.A. generated and interpreted 3D confocal imaging data. M.S. conducted *in vivo* experiments. A.B. and B.S. conducted, analyzed, and interpreted pharmacoscopy data. M.M. conducted and analyzed retroviral transduction. F.P. and F.S. performed and evaluated H&E stainings. J.F., C.W., and S.P. generated pseudouridine mRNA. M.W. interpreted data and wrote the manuscript.

## Declaration of interests

S.P. reports that a patent application has been filed pending. M.W. reports grants from the University of Zurich during the conduct of the study; M.W. also reports grants from AbbVie, Adastra, Apogenix, Merck Sharp & Dohme, Merck (EMD), Novocure, and Quercis, as well as personal fees from AbbVie, Adastra, Bristol Myers Squibb, Celgene, Medac, Merck Sharp & Dohme, Merck (EMD), Nerviano Medical Sciences, Novartis, Orbus, Philogen, Roche, Tocagen, and Y-mAbs outside the submitted work. T.W. reports grants from the University of Zurich, Swiss Cancer Research, Betty and David Koetser Foundation, Promedica Foundation, and Helmut Horten Foundation during the conduct of the study, as well as personal fees from Philogen outside the submitted work. C.N.A. reports grants from the CRPP ImmunoCure of the University of Zurich.

## STAR★Methods

### Key resources table

**Table:** 

REAGENT or RESOURCE	SOURCE	IDENTIFIER
**Antibodies**		

Anti-hCD3-PE	BioLegend	Cat#300308; RRID: AB_314043
Anti-hCD8-PerCP	BioLegend	Cat#301030; RRID: AB_893425
Anti-hγδTCR-APC	BioLegend	Cat#331212; RRID: AB_1089215
Anti-h/mCD11b-APC/Cy7	BioLegend	Cat#101225; RRID: AB_830641
Anti-hHLA-A,B,C-APC	BioLegend	Cat#311417; RRID: AB_493669
Anti-mNKG2D-APC	BioLegend	Cat#130211; RRID: AB_1236372
Anti-mCD45.1-AF488	BioLegend	Cat#110718; RRID: AB_492862
Anti-mCD45.2-PE	BioLegend	Cat#109807; RRID: AB_313444
Anti-mCD11b-BV605	BioLegend	Cat#101257; RRID: AB_11126744
Anti-mCD206-APC	BioLegend	Cat#141708; RRID: AB_10896057
Anti-mCD86-PE	BioLegend	Cat#305405; RRID: AB_314525
Anti-mCD86-BV650	BioLegend	Cat#105036; RRID: AB_11126147
Anti-mH2-Db-APC	BioLegend	Cat#111514; RRID: AB_2565862
Anti-mCD335-BV510	BioLegend	Cat#137623; RRID: AB_2563290
Anti-mCD11b-BV605	BioLegend	Cat#101257; RRID: AB_11126744
Anti-mCD206-PE/Cy7	BioLegend	Cat#141719; RRID: AB_2562247
Anti-mCD45.2-PE/Dazzle	BioLegend	Cat#109845; RRID: AB_2564176
Anti-mNKG2D-APC	BioLegend	Cat#130211; RRID: AB_1236372
Anti-hCD4-FITC	BD Biosciences	Cat#555346; RRID: AB_395751
Anti-hCD56-APC	BD Biosciences	Cat#555518; RRID: AB_398601
Anti-mCD8-BV786	BD Biosciences	Cat#563332; RRID: AB_2721167
Anti-mRae1-BV711	BD Biosciences	Cat#748077; RRID: AB_2872538
Anti-mCD3-PerCP/Cy5.5	BD Biosciences	Cat#560527; RRID: AB_1727463
Anti-mCD335-PE	BD Biosciences	Cat#560757; RRID: AB_1727466
Anti-hCD34(RQR8)-PE	Thermo Fisher Scientific	Cat#MA1-10205; RRID: AB_11152571
Anti-hMICA/MICB	eBioscience	Cat#12-5788-42; RRID: AB_10854117
Anti-hNKG2D-APC	eBioscience	Cat#17-5878-81; RRID: AB_469462
Anti-hS100B-AF555	Abcam	Cat#ab215989
Anti-hNestin-PE	BioLegend	Cat#656806; RRID: AB_2566381
Anti-hCD45-AF647	BioLegend	Cat#368538; RRID: AB_2716028
Anti-hEndomucin (V.7C7)	Santa Cruz Biotechnology	Cat#Sc-65495
Donkey-*anti*-rat AF647	Thermo Fisher Scientific	Cat#A48272; RRID: AB_2893138
InVivoMAb anti-mouse CD3ε (clone 145-2C11)	BioXCell	Cat#BE0001-1; RRID: AB_1107634
InVivoMAb anti-mouse CD28 (clone 37.51)	BioXCell	Cat#BE0015-1; RRID: AB_1107624

**Bacterial and virus strains**		

Retrovirus MSGV-1D3-28Z	Addgene	Cat#107226

**Biological samples**		

Human glioblastoma samples	Neurosurgery Department of the University Hospital of Zurich	https://www.usz.ch/en/department/neurosurgery/

**Chemicals, peptides, and recombinant proteins**		

Recombinant Murine IL-2	Peprotech	Cat#212-12
Recombinant Murine IL-15	Peprotech	Cat#210-15
Recombinant Human IFN-γ	Peprotech	Cat#300-02
Recombinant Human TGF-β1 (HEK293 derived)	Peprotech	Cat#100-21
Recombinant Human TGF-β2 (HEK293 derived)	Peprotech	Cat#100-35B
Animal-Free Recombinant Human EGF	Peprotech	Cat#AF-100-15-1
Recombinant Human FGF-basic (154 a.a.)	Peprotech	Cat#100-18B
Recombinant Human M-CSF (carrier-free)	Biolegend	Cat#574804
Recombinant Human GM-CSF (carrier-free)	Biolegend	Cat#572904
Dynabeads™ Human T-Activator CD3/CD28 for T cell Expansion and Activation	Thermo Fisher Scientific	Cat#11131D
Formaldehyde 16% Sol. em Grade 10x10mL	Electron Microscopy Sciences	Cat#15710
ACK lysis buffer	Thermo Fisher Scientific	Cat#A1049201
Percoll	Sigma-Aldrich	Cat#17-0891-02
Collagenase from clostridium histolyticum	Sigma-Aldrich	Cat#C5138-1G
Deoxyribonuclease I from bovine pancreas	Sigma-Aldrich	Cat#DN25-100MG
Retro-Concentin	Systems Biosciences	Cat#SBI-RV100A-1
RapiClear 1.52	Sunjin Lab	Cat#RC152002:100mL
Fugene 6 transfection reagent	Promega	Cat#E2693
RetroNectin® Recombinant Human Fibronectin Fragment	Takara	Cat#T100B
Polybrene	Santa Cruz	Cat#sc-134220

**Critical commercial assays**		

EasySep Mouse NK Cell Isolation Kit	STEMCELL Technologies	Cat#19855
EasySep™ Release Human CD3 Positive Selection Kit	STEMCELL Technologies	Cat#17751
Pan T cell Isolation Kit II, mouse	Miltenyi Biotec	Cat#130-095-130
EasySep™ Human CD14 Positive Selection Kit II	STEMCELL Technologies	Cat#17858
PKH26 Red Fluorescent Cell Linker Kit	Sigma-Aldrich	Cat#PKH26GL-1KT
Zombie Violet Fixable Viability Kit	Biolegend	Cat#423114
Chromium Next GEM Single Cell 3ʹ GEM, Library & Gel Bead Kit v3.1, 4 rxns	10x Genomics	Cat#PN-1000128
3′ CellPlex Kit	10x Genomics	Cat#PN-1000261
IFN gamma Mouse Uncoated ELISA Kit with Plates	Thermo Fisher Scientific	Cat#88-7314-22

**Deposited data**		

scRNA-seq data	This paper	GEO: GSE283049
scRNA-seq code	This paper	Zenodo: https://doi.org/10.5281/zenodo.14230743

**Experimental models: Cell lines**		

LN-229	Dr. N. de Tribolet	N/A
ZH-161	Neurology Department of the University Hospital of Zurich	N/A
CT-2A	Merck	Cat#SCC194
GL-261	National Cancer Institute	N/A
SB-28	Leibniz-Institute German Collection of Microorganisms and Cell Cultures (DSMZ)	Cat#ACC 880
L929	Dr. Tomasz Rygiel at the Department of Immunology, Medical University of Warsaw	N/A
E0771-BrM	Dr. Manuel Valiente at the Spanish National Cancer Research Center (CNIO) of Madrid	N/A
LLC-BrM	Dr. Manuel Valiente at the Spanish National Cancer Research Center (CNIO) of Madrid	N/A
Platinum-E (Plat-E) Retroviral Packaging Cell Line	Cell Biolabs	N/A
K562-mbIL21 feeder cells	Dr. Obinna Chijioke at Institute of Experimental Immunology of University of Zurich	N/A
A20	Dr. Christian Pellegrino at Department of Medical Oncology and Hematology of University Hospital Zurich	N/A

**Experimental models: Organisms/strains**		

C57BL/6J	Janvier Labs	Cat#SC-C57N-F
C57BL/6J 45.1	Own breeding	N/A

**Software and algorithms**		

CellProfiler (2.2.0 and 4.2.1)	https://cellprofiler.org/	N/A
FlowJo (Tree Star)	https://www.flowjo.com/solutions/flowjo	N/A
GraphPad	https://www.graphpad.com/features	N/A
CellRanger version 7.1.0	https://www.10xgenomics.com/support/software/cell-ranger/latest	N/A
R version 4.3.1	https://www.r-project.org/	N/A
scRNA-seq code	This paper	Zenodo: https://doi.org/10.5281/zenodo.14230743

**Other**		

96-well ViewPlates	PerkinElmer	Cat#6005182
Corning®-Zellsieb pore size 70 μm	Sigma-Aldrich	Cat#CLS431751

### Experimental model and study participant details

#### Animals

All experiments were done in accordance with the guidelines of the Swiss federal law on animal protection and were approved by the cantonal veterinary office. Female C57BL/6^CD45.2^ (#SC-C57N-F) mice of 6–12 weeks of age were purchased from Janvier Labs. C57BL/6^CD45.1^ mice of both sexes were bred in pathogen-free facilities at the University of Zurich (Zurich, Switzerland).

#### Human participants

Glioblastoma tissue samples of 10 randomly selected patients were obtained from the Neurosurgery Department of the University Hospital of Zurich after informed consent (Data S1) and under the approval of the Institutional Review Board with the ethical approval number (BASEC-Nr: 2019-01721). One female and nine male patients with an age of 44–78 years were included of which eight patients had a primary tumor and did not receive any previous treatment, one had a preceding surgery, and one received marizomib, temozolomide and radiotherapy. Each sample represents one patient and was subjected to each experimental condition.

#### Cell lines

GL-261 cells were obtained from the National Cancer Institute and SB-28 cells (#ACC 880) were obtained from the Leibniz-Institute German Collection of Microorganisms and Cell Cultures (DSMZ). GL-261 iRFP720 and GL-261 tdTomato cells were generated as described.^31^ The human malignant glioma cell line LN-229 was kindly provided by Dr. N. de Tribolet. GL-261, CT-2A, SB-28 and LN-229 were maintained in Dulbecco’s Modified Eagle Medium (DMEM), containing 2 mM L-glutamine (Gibco Life Technologies), 1% penicillin/streptomycin and 10% fetal calf serum (FCS, Gibco Life Technologies). ZH-161 was established from freshly dissected tumor tissue and maintained in neurobasal medium (Gibco Life Technologies) supplemented with 20 ng/mL fibroblast growth factor 2 and EGF (PeproTech, Rocky Hill, PA), 20 μL/mL B-27 (Gibco Life Technologies) and 2 mM L-glutamine. The A20 cell line was maintained in RPMI medium containing 2 mM L-glutamine (Gibco Life Technologies), 1% penicillin/streptomycin, 10% fetal calf serum (FCS, Gibco Life Technologies) and 0.05 mM 2-mercaptoethanol (Sigma-Aldrich). All cell lines were regularly tested negative for mycoplasma by PCR.

#### Primary cell cultures

Primary mouse T cells, NK cells and macrophages were differentiated and expanded from splenocytes or bone marrow from C57BL/6CD45.1 or C57BL/6CD45.2 mice as previously described (Look et al. 2023). If not indicated otherwise, mouse T cells were used on day 5, NK cells on day 7 and macrophages on day 5 of differentiation for *in vitro* and *in vivo* experiments. For long-term expansion after retroviral transduction and Sleeping Beauty transposition mouse T cells were kept in RPMI medium containing 2 mM L-glutamine (Gibco Life Technologies), 1% penicillin/streptomycin, 10% fetal calf serum (FCS, Gibco Life Technologies), 0.05 mM 2-mercaptoethanol (Sigma-Aldrich) and supplemented with 0.1 mmol/L non-essential amino acids (Gibco Life Technologies), 10 ng/mL human IL-7 and 10 ng/mL human IL-15 (Miltenyi) from day 3 after isolation.

Primary human immune cells were expanded from PBMCs of healthy donors after informed consent and institutional approval (BASEC-Nr.: 2019–02027) and cultivated in RPMI medium containing 2 mM L-glutamine (Gibco Life Technologies), 1% penicillin/streptomycin and 10% fetal calf serum (FCS, Gibco Life Technologies). Lymphocyte medium was additionally supplemented with 0.05 mM 2-mercaptoethanol (Sigma-Aldrich). PBMCs were isolated using ROTI-Sep 1077 (Carl Roth, #0642.2) and centrifugation at 700*g* without break for 30 min. Monocytes were isolated from PBMCs using the EasySep Human CD14 Positive Selection Kit II (Stemcell Technologies, #17858) and seeded with 0.5 × 106 cells/ml on non-adherent petri dishes. The medium was either supplemented with 20 ng/mL GM-CSF (day 0–7) and 20 ng/mL IFNγ (day 5–7) or 50 ng/mL M-CSF (day 0–7). Differentiated macrophages were starved for 12 h in medium with 0.1% FCS and without cytokines before functional use. Human T cells and NK cells were expanded from CD3^+^ and CD3^−^fractions separated from PBMCs using the EasySep Release Human CD3 Positive Selection Kit (Stemcell Technologies, #17751). Enriched CD3^+^ cells were kept at a density of 1 × 106 cells/ml and activated with Dynabeads Human T-Activator CD3/CD28 for T cell Expansion and Activation (Thermo Fisher, # 11131D) for three days. Medium was supplemented with 100 U/ml IL-2 throughout culture. Expanded T cells were used for functional assays 12–14 days after isolation. Enriched CD3^−^cells were co-cultured with irradiated (100 Gy) K562-mbIL21 feeder cells at a 1:1 ratio and medium supplemented with 200 U/ml IL-2 throughout culture. NK cells were kept at a density of 0.5–1 x 106 cells/ml. Negative selection for CD3^−^cells and co-culture with feeder cells was repeated 7 days after isolation. Expanded NK cells were used for functional assays 14 days after isolation.

### Method details

#### *In vitro* transcription of mRNA

The mouse and human NKG2D CAR constructs have been described previously.^16^^,^^20^^,^^23^ Additionally, a Furin-T2A cleavage site followed by the RQR8 gene was integrated downstream of the CAR sequences. In NKG2D CARΔ(CD3ζ) constructs the functional CD3ζ sequence was removed. The mRNA encoding mIL12 was obtained from BioNTech. Human mRNA sequences for IFNα2, IL-12A and IL-12B, connected via a (G4S)3 linker, were derived from NCBI with NM_000605.4, NM_001397992.1 and NM_002187.3, respectively. Synthetic m RNA (5′ CleanCap, fully 1-methyl Pseudouridine, both from Trilink) was synthesized from DNA matrices (synthetic genes ordered at Twist Bioscience) using HiScribe (NEB Biolabs) and purified by LiCl precipitation as described previously.^34^ The transcripts were resuspended at 1–2 mg/mL in pure water. Size and integrity were verified using MOPS-formaldehyde agarose gel electrophoresis. Functionality of *in vitro* transcribed mRNAs was confirmed by transfection of lymphocytes or HEK293T cells and subsequent analysis of protein expression by flow cytometry or ELISA.

#### CAR immune effector cell generation

Mouse and human immune cells were electroporated using a NEON transfection system (Invitrogen). Electroporation parameters were set to a voltage of 1600 V, 10 ms, 3 pulses (mouse and human lymphocytes); 1600 V, 10 ms, 2 pulses (mouse macrophages) or 1500 V, 10 ms, 3 pulses (human macrophages).

Mouse and human mRNAs coding for chimeric antigen receptors, cytokines or ZsGreen were used at different concentrations for electroporation indicated in Table S1. Mock-electroporated cells were electroporated without mRNA and served as a control. Sleeping Beauty transposition was performed by electroporation with 6 μg mRNA coding for the Sleeping Beauty transposase SB100X and 4 μg of the vector pT4 carrying the mouse NKG2D-Furin/T2A-RQR8 transgene (Figure 1F). Following electroporation, the cells were kept in antibiotics-free medium and used for experiments within a few hours.

The sequence of mouse NKG2D-Furin/T2A-RQR8 (Figure 1F) was cloned into the retroviral backbone pMSGV. MSGV-1D3-28Z was a gift from James Kochenderfer and Steven Rosenberg (Addgene plasmid #107226).^35^ The empty backbone was used to generate mock-transduced T cells. Retrovirus was produced by transfection of 10^7^ Platinum-E cells (Cell Biolabs) with 75 μL of Fugene 6 (Promega) and 25 μg of the according plasmid for 24 h. After 48 and 72 h supernatants were harvested and concentrated with Retro-Concentin (Systems Biosciences). Concentrated retrovirus was added to plates coated with 24 μg/mL RetroNectin (Takara) by centrifugation for 2 h. T cell plates were additionally coated with 5 μg/mL αCD3 and 2 μg/mL αCD28 (BioXcell). T cells were isolated using a T cell isolation kit (Miltenyi, #130-095-130) and transduced 24 h later by 20 min spinoculation to the retroviral-coated wells, followed by 48 h of incubation. Similarly, NK cells and macrophages were transduced on day 3 and day 4 of expansion, respectively.

#### Lentiviral transduction of mouse NK cells

Mouse NK cells were transduced on day 4 of expansion with varying concentrations of pLenti-CMV-empty or pLenti-CMV-GFP vector in the presence of 8 μg/mL Polybrene. Plates were spinoculated for 1.5 h at 1000 g at 32°C, followed by medium exchange 24 h later. Transfection efficiency was quantified 48 h after spinoculation using flow cytometry.

#### Live-cell imaging

Immunofluorescent imaging of ZsGreen mRNA transfected immune cells and live-cell imaging were performed using a MuviCyte microscope (PerkinElmer). For live-cell imaging 10,000 GL-261 tdTomato cells were pre-seeded (for macrophages co-seeded) on 96-well ViewPlates (PerkinElmer, #6005182) and co-cultured with ZsGreen mRNA or ZsGreen mRNA plus CAR mRNA or ZsGreen plus CARΔ(CD3ζ) mRNA transfected immune cells. Pictures were taken every 10 min. Tumor cell confluence was analyzed using the “Wound Healing” pipeline and immune cell fluorescence after ZsGreen mRNA transfection using the “Human cells” pipeline from CellProfiler (4.2.1) software. Videos were rendered using ImageJ software.

#### Antibodies and flow cytometry

A detailed list of antibodies used for flow cytometry can be found in the key resources table. Acquisition was performed on a BD FACSVerse Analyzer or BD LSR II Fortessa 4L and data were analyzed with FlowJo (Tree Star). Tumor-infiltrating immune cells were subclassified in CD11b^+^CD45^high^ myeloid cells, CD11^+^CD45^medium^ microglia, CD45^+^CD11b^+^CD3^+^Nkp46^−^CD8α^+^ T cells and CD45^+^CD11b^−^CD3^−^CD335^+^ NK cells.

#### *In vitro* co-culture assays

For co-culture experiments, 25,000 mouse or human tumor cells were stained with PKH26 (Sigma-Aldrich) and pre-seeded (for macrophages co-seeded) into 96-well plates. Afterward, up to 250,000 immune cells were added in the presence or absence of 50 ng/mL TGFβ1 or TGFβ2 and tumor cell viability was assessed 24 h later with the Zombie Violet Fixable Viability Kit (BioLegend) and flow cytometry. Target cell lysis was determined as the percentage of death in the population of labeled target cells after subtraction of background lysis. Co-cultures with macrophages were additionally stained for anti-CD11b and tumor cell numbers quantified using high flow rate for 1 min.

#### Mice and animal experiments

For intracranial tumor implantation, GL-261 (2 × 10^4^), GL-261 iRFP720 (4 × 10^4^), SB-28 (4 × 10^4^), E0771-BrM (6 × 10^4^) cells were stereotactically implanted into the right striatum. Mice were observed daily for the development of symptoms. If not indicated otherwise, adoptive cell transfer of mock, CAR or multifunctional CAR immune effector cells was performed as following: 5 × 10^6^ cells were intravenously injected via the tail vein on day 5 and 10 after tumor cell implantation; 2 × 10^6^ cells were locally injected into the tumor on day 5 and 10 after tumor cell implantation. Blood was collected 12 days after tumor cell implantation and clinical parameters analyzed by the Veterinary Laboratory at University of Zurich using a Roche Cobas c501 analyzer. Isolation of tumor cells and tumor-infiltrating immune cells was done as described before,^36^ briefly mice were perfused with Dulbecco’s Phosphate Buffered Saline (DPBS) and the tumor-bearing brain hemisphere was collected in Roswell Park Memorial Institute (RPMI) medium containing 0.4 mg/mL Collagenase IV and 0.1 mg/mL DNase I (both Sigma-Aldrich). The tissue was then incubated for 40 min at 37°C and further homogenize using an 18G needle. Myelin was removed using a 30% Percoll centrifugation step (Sigma-Aldrich). For isolation of brains for 3 dimensional (3D) confocal microscopy, mice were deeply anesthetized, perfused with cold DPBS followed by a cold DPBS solution containing 4% paraformaldehyde (PFA, Electron Microscopy Sciences). After isolation, brains were incubated in 4% PFA over night at 4°C and stored in DPBS with 0.01% sodium azide (NaN_3_) at 4°C until embedding.

#### 3D confocal microscopy

Brains were embedded in 5% low gelling temperature agarose, at least 1 h before processing. Samples were then sectioned with a fully automated vibratome (Leica VT1200 S, Leica Microsystems, Germany) and the following settings: speed 0.3 mm/s, feed 245 μm, amplitude 1.35 mm. Brain slices were subsequently immunostained, following an initial blocking step (0.2% Triton X-100 and 10% donkey serum in PBS) overnight at 4°C, with a primary antibody against endomucin (Table S2) diluted in blocking buffer, for two days at 4°C. Primary antibodies were washed using 0.2% Triton X-100 in PBS, and secondary antibody staining was performed for another two days at 4°C. Immunostained slices were then washed once more and incubated in RapiClear 1.52 (Sunjin Lab) overnight at 4°C to ensure tissue clearing. Confocal microscopy was then performed with a Stellaris 5 upright microscope (Leica, Germany) and image analysis was performed using Imaris 10.0.0 (Bitlplane AG) software. The tumor surface mask was created using the magic wand tool for every tenth slice in the DAPI channel. CAR immune effector cells were first annotated manually using spot identification in render mode and ZsGreen plus DAPI overlay. Based on the lowest median ZsGreen intensity, a threshold was defined and all cells in the tumor mask automatically annotated. The endothelium was segmented using surface identification with endomucin channel as source, together with the quality filter and voxel filtering (min 1000). Cumulative values of CAR immune effector cells outside of the endothelium surface and automatically generated random simulation values were exported from Imaris in vantage mode at spatial view. Exported data was plotted as cumulative distribution function (CDF) or complete spatial random (CSR) plots in GraphPad PRISM. To study CAR immune effector cell infiltration with respect to the endothelium, two-sample Kolmogorov-Smirnov test was used to analyze significance between CDF and CSR plots. Cell density maps were created to qualitatively assess the overall distribution of CAR T cells, CAR NK cells and CAR macrophages. These 2D maps were generated using an adaptation of the kernel-based density estimation method, which employs the cells’ coordinates as 3D Gaussian kernels, which are later averaged across the axial dimension as reported.^37^ The tissue map borders depict the tumor surface mask.

#### Histological evaluation

Brain, liver and spleen from euthanized mice (Figures 4J and S6A–S6C) were fixed in 10% neutral buffered formalin for 48 h prior to embedding in paraffin. Three-micrometer thick sections were stained with haematoxylin and eosin (H&E). Pathological evaluation was performed independently and in a blinded manner by two board-certified veterinary pathologists.

#### ELISA

Two days and five days after CAR immune cell treatment supernatant of dissociated mouse tumor-bearing hemispheres and mouse blood plasma were collected and frozen at −80°C. IFNγ concentration of undiluted supernatant and 1:4 diluted plasma were quantified using IFNγ Mouse Uncoated ELISA Kit (Thermo Fisher, #88-7314-22).

#### Single-cell RNA-Sequencing

For scRNA-seq mice received a local injection of 2 × 10^6^ CAR immune effector cells on day 7 after GL-261 tumor cell implantation and brains were isolated 5 days later. Tumor tissues were excised, placed in ice-cold HBSS supplemented with glucose (10 mM) and HEPES (10 mM) and mechanically dissociated using glass shearing with a 10-mL Potter-Elvehjem pestle and glass-tube homogenizer (Merck). The resulting suspension was passed through a 70-μm cell strainer (BD Biosciences). The cells were incubated with FC blocking (BD Biosciences). Simultaneously, 20 μL of Cell Multiplexing Oligo (CMO) lipid solution (3′ CellPlex Kit, 10X Genomics) was added for multiplexing for 20 min. Afterward, cells were washed with flow cytometry buffer and incubated for 20 min with anti-CD45 or anti-CD45.2 antibodies (BioLegend). Cell sorting was performed on a MoFlo Astrios (Beckman Coulter). A total of 50,000 cells were sorted per sample and analyzed using 10X Genomics 3′ single-cell mRNA sequencing (v3.1 chemistry), resulting in a final yield of approximately 10,000 sequenced cells per reaction.

Libraries were prepared following the manufacturer’s instructions and sequenced on an Illumina NextSeq 550 machine. The resulting fastq files were demultiplexed and aligned to a mouse reference transcriptome (refdata-gex-mm10-2020-A provided on the 10X Genomics website) using the “cellranger multi” function within the CellRanger version 7.1.0 software.

Single-cell data analysis was performed in R version 4.3.1 (2023-06-16). Cell doublets were identified and excluded using a combination of the scDblFinder version 1.14.0 and SingleCellExperiment version 1.24.0 packages. Cells with more than 5 percent mitochondrial gene content and 5,000 or more detected genes were excluded. Downstream analysis of *n* = 33,345 cells was conducted in Seurat version 5.0.1.^38^ Gene Expression modules were quantified using the UCell package version 2.4.0.^39^ Cluster abundance enrichment analysis was done using hypergeometric testing in R. The local abundance of the treatment associated with each cell was visualized using the ggpointdensity package (version 0.1.0). Pseudotime and gene ontology analyses were performed using monocle3 (version 1.3.4) and ClusterProfiler (version 4.8.3) packages.^40^^,^^41^ Significantly enriched genes in monocle were identified based on adjusted *p* values and Moran’s I parameters assessed with the graph_test() function of monocle3. For plotting, we utilized tidyverse version 2.0.0 and ComplexHeatmap version 2.16.0^42^^,^^43^.

#### Pharmacoscopy

Glioblastoma tissue samples of 10 patients were rinsed with PBS and cut into small fragments. The tissue fragments were further dissociated with a digestion mix containing DMEM medium (#41966029) with 10% FBS (#10270106) and 1% Pen-strep (#15140122), all from Gibco, Collagenase IV (1 mg/ml, Sigma Aldrich) and DNaseI (0.1 mg/ml Sigma Aldrich) for 45 min at 37°C using the MACS Gentle Dissociator (Miltenyi Biotec, 130-096-427). The resulting suspension was passed through a 18G needle, strained through a 70 μm Corning cell strainer (Sigma-Aldrich, CLS431751) and washed with PBS containing 2 mM EDTA (Invitrogen). Myelin was removed by gradient centrifugation with 30% Percoll (Sigma-Aldrich, #17-0891-02) at 1592 g without break and red blood cells were removed by incubation with ACK lysis buffer (Thermo Fisher Scientific, A1049201) for 5 min at RT. Single cell suspensions were frozen down in Bambanker freezing medium and were kept in liquid nitrogen until required.

Patient single cell suspensions were seeded together with CAR effector cells in a 1:1 ratio (8000 cells each) into clear-bottom, tissue-culture treated, CellCarrier-384 Ultra Microplates (PerkinElmer, #6057300). As a control, patient single cell suspensions were treated with PBS. After 24 h of incubation at 37°C, 5% CO_2_ the cells were fixed with 4% PFA (Sigma-Aldrich) for 15 min at RT, blocked with PBS containing 5% FBS, 0.1% Triton X- and DAPI (4 μg/mL, #422801, Biolegend) for 1 h at RT and stained with PE anti-NESTIN (Biolegend, #656806, 1:150), self-conjugated Alexa Fluor 555 anti-S100B (Abcam, ab215989, 1:1000) and Alexa Fluor 647 anti-CD45 (Biolegend, #368538, 1:300) overnight at 4°C. The 384-well plates were imaged with the Opera Phenix automated spinning-disk confocal microscope at 20× magnification (PerkinElmer, HH14000000). Cell segmentation was performed based on the DAPI channel using CellProfiler 2.2.0 and the downstream image analysis was carried out with MATLAB R2021b. Marker-positive cells were determined for each condition by using a linear threshold of the histograms of each channel. Five replicates were used per treatment condition and eight replicates for the PBS control group. Relative glioblastoma cell fractions were calculated as the mean fraction of NESTIN/S100B-positive viable cells after drug treatment divided by the mean fraction of NESTIN/S100B-positive viable cells detected in PBS-containing control wells.

### Quantification and statistical analysis

Data are presented as means of triplicates and SD unless otherwise indicated. Statistical analyses were performed in GraphPad Prism using t-Test or one-way analysis of variance (ANOVA) as indicated and correction for multiple comparisons using the Dunnett or Tukey method. Kaplan Meier survival analysis was performed to assess survival differences among the treatment groups and *p* values were calculated with the log rank test. Significance was concluded at ∗*p* < 0.05, ∗∗*p* < 0.01, ∗∗∗*p* < 0.001.

## Supplemental information

Document S1. Figures S1–S8 and Table S1

Data S1. Consent form, related to Figure 6

Document S2. Article plus supplemental information
