# Supplementary material for: CAR T cells, CAR NK cells, and CAR macrophages exhibit distinct traits in glioma models but are similarly enhanced when combined with cytokines
Source: Cell Rep Med. 2025 Jan 30;6(2):101931. doi: 10.1016/j.xcrm.2025.101931 (PMC11866521; doi:10.1016/j.xcrm.2025.101931)
Supplement: Document S1. Figures S1–S8 and Table S1 [file mmc1.pdf]

Cell Reports Medicine, Volume *6*

Supplemental information

CAR T cells, CAR NK cells, and CAR macrophages exhibit distinct traits in glioma models but are similarly enhanced when combined with cytokines

Thomas Look, Roman Sankowski, Manon Bouzereau, Serena Fazio, Miaomiao Sun, Alicia Buck, Niklas Binder, Maximilian Mastall, Francesco Prisco, Frauke Seehusen, Julia Frei, Conrad Wyss, Berend Snijder, Cesar Nombela Arrieta, Michael Weller, Steve Pascolo, and Tobias Weiss

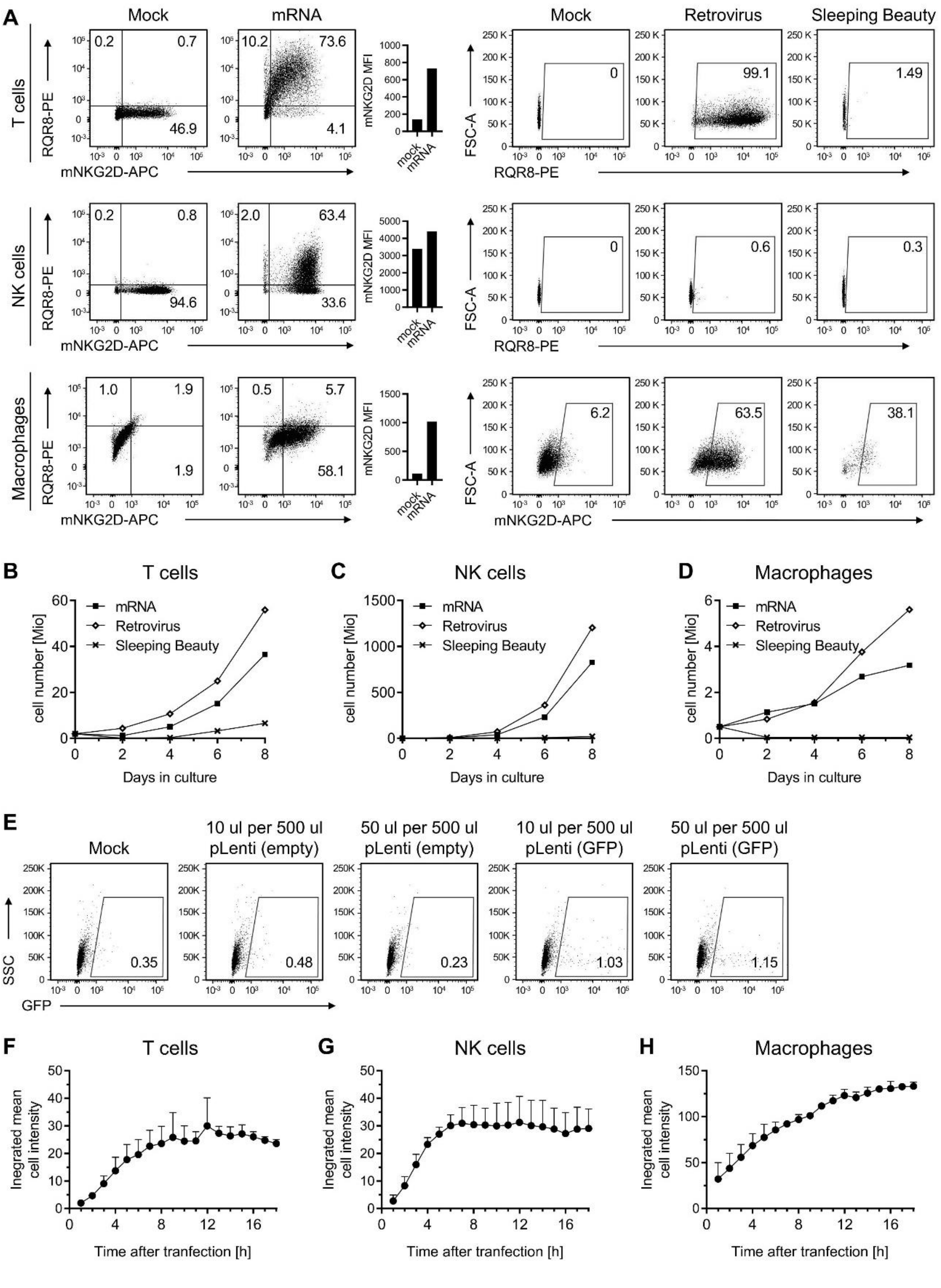

A
Mock
mRNA
Mock
Retrovirus
Sleeping Beauty
T cells
NK cells
Macrophages
RQR8-PE
mNKG2D-APC
mNKG2D MFI
mock
mRNA
FSC-A
B
T cells
C
NK cells
D
Macrophages
mRNA
Retrovirus
Sleeping Beauty
cell number [Mio]
Days in culture
E
10 ul per 500 ul pLenti (empty)
50 ul per 500 ul pLenti (empty)
10 ul per 500 ul pLenti (GFP)
50 ul per 500 ul pLenti (GFP)
SSC
GFP
F
G
H
Inegrated mean cell intensity
Time after tranfection [h]

Figure S1: mRNA proves superior in generating functional CAR immune effector cells. Related to Figure 1. (**A-D**) Mouse T cells, NK cells or macrophages were transfected with mRNA, a retrovirus or a pT4 Sleeping Beauty vector coding for mouse NKG2D-Furin/T2A-RQR8 (CAR) (**A**) Flow cytometry analysis of mouse NKG2D or RQR8 expression one day after transfection. (**B-D**) Cell numbers of T cells (**B**), NK cells (**C**) and macrophages (**D**) starting from the day of transfection. (**E**) Mouse NK cells were transduced with pLenti-CMV-empty or pLenti-CMV-GFP vector for 24 hours and subsequently incubated for another 24 hours in fresh medium. Flow cytometry quantification of GFP expression is shown. (**F-H**) Mouse T cells (**F**), NK cells (**G**) or macrophages (**H**) were transfected with ZsGreen mRNA and cell fluorescence was analyzed hourly using live cell imaging. Data are represented as mean + SD based on n = 3 FOVs. FOV, field of view.

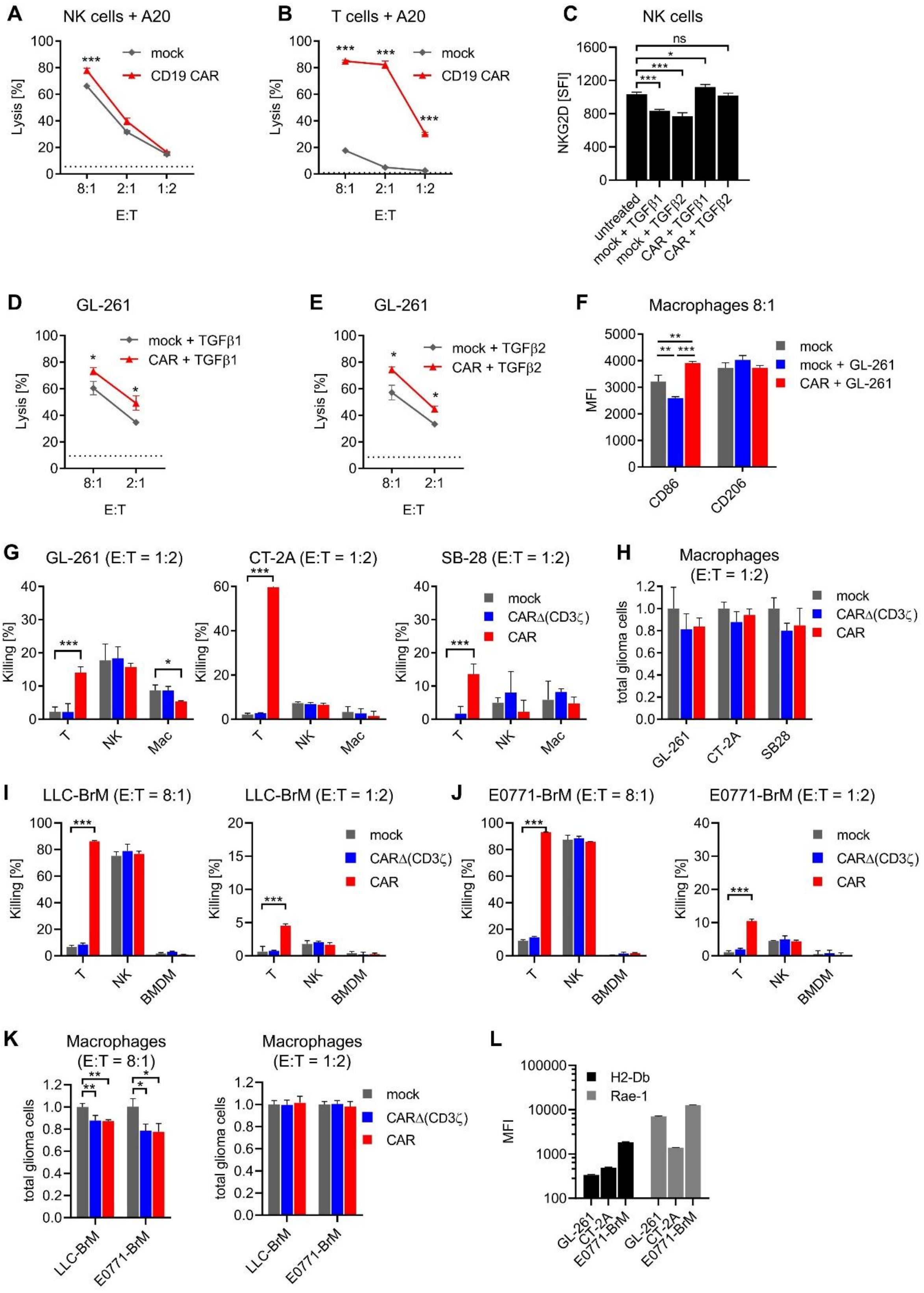
A
NK cells + A20
mock
CD19 CAR
Lysis [%]
E:T
8:1
2:1
1:2
B
T cells + A20
C
NK cells
NKG2D [SFI]
untreated
mock + TGFβ1
mock + TGFβ2
CAR + TGFβ1
CAR + TGFβ2
ns
D
GL-261
mock + TGFβ1
CAR + TGFβ1
E
GL-261
mock + TGFβ2
CAR + TGFβ2
F
Macrophages 8:1
mock
mock + GL-261
CAR + GL-261
MFI
CD86
CD206
G
GL-261 (E:T = 1:2)
CT-2A (E:T = 1:2)
SB-28 (E:T = 1:2)
Killing [%]
T
NK
Mac
mock
CARΔ(CD3ζ)
CAR
H
Macrophages
(E:T = 1:2)
total glioma cells
GL-261
CT-2A
SB28
I
LLC-BrM (E:T = 8:1)
LLC-BrM (E:T = 1:2)
BMDM
J
E0771-BrM (E:T = 8:1)
E0771-BrM (E:T = 1:2)
K
Macrophages
(E:T = 8:1)
Macrophages
(E:T = 1:2)
LLC-BrM
E0771-BrM
L
H2-Db
Rae-1
MFI
GL-261
CT-2A
E0771-BrM

Figure S2: mRNA proves superior in generating functional CAR immune effector cells. Related to Figure 1. (**A-B**) Mock or CD19 CAR mRNA-transfected mouse NK cells (**A**) or T cells (**B**) where co-cultured with A20 lymphoma cells at different E:T ratios for 24 hours and A20 cell killing quantified using flow cytometry (mean +/- SD of $n = 3$, paired t test with *$P < 0.05$; **$P < 0.01$, ***$P < 0.001$). (**C-E**) Mock or CAR mRNA-transfected mouse NK cells were cultured alone or together with GL-261 glioma cells in the presence of 50 ng/ml TGFβ1 or TGFβ2. (**C**) Flow cytometry quantification of NKG2D surface expression on mouse NK cells 12 hours after incubation with TGFβ1 or TGFβ2 (mean + SD of $n = 3$, one-way ANOVA with *$P < 0.05$; **$P < 0.01$; ***$P < 0.001$). (**D-E**) Glioma cell lysis after co-culture (mean +/- SD of $n = 3$, paired t test with *$P < 0.05$). (**F**) Mock or CAR mRNA-transfected mouse macrophages were co-cultured with GL-261 glioma cells for 24 hours. Flow cytometry quantification of CD86 and CD206 surface expression are shown ($n = 3$, one-way ANOVA with *$P < 0.05$; **$P < 0.01$; ***$P < 0.001$). (**G-K)** Flow cytometry quantifications of GL-261, CT-2A, SB-28, LLC-BrM or E0771-BrM tumor cells that were co-cultured at an E:T ratio of 8:1 or 1:2 for 24 hours with mouse immune cells that were mock transfected or transfected with mRNA coding for CAR or CARΔ(CD3ζ). (**G, I, J**) Glioma cell lysis and (**H, K**) total remaining glioma cells after co-culture are shown (mean + SD of $n = 3$, one-way ANOVA with *$P < 0.05$; **$P < 0.01$; ***$P < 0.001$). (**L**) Flow cytometry quantification of absolute H2-Db and Rae-1 surface expression on GL-261, CT-2A and E0771-BrM tumor cells are shown (mean + SD of $n = 3$).

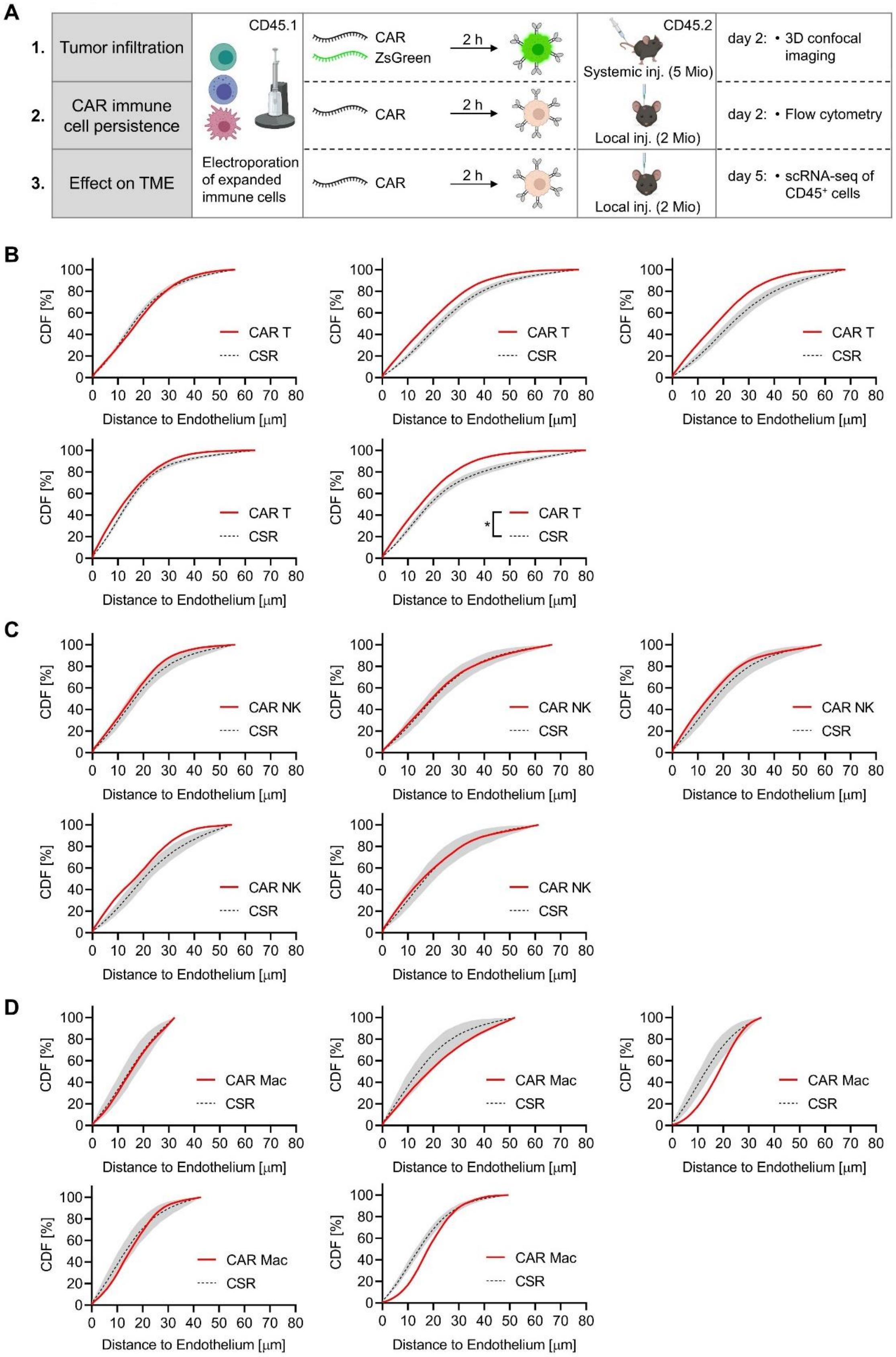
A
1. Tumor infiltration
2. CAR immune cell persistence
3. Effect on TME
CD45.1
Electroporation of expanded immune cells
CAR
ZsGreen
2 h
CD45.2
Systemic inj. (5 Mio)
Local inj. (2 Mio)
Local inj. (2 Mio)
day 2: • 3D confocal imaging
day 2: • Flow cytometry
day 5: • scRNA-seq of CD45⁺ cells
B
CDF [%]
Distance to Endothelium [µm]
CAR T
CSR
*
C
CAR NK
CSR
D
CAR Mac
CSR

Figure S3: Approaches to study CAR immune effector cells in vivo and tumor-infiltrative capacities of CAR immune effector cells. Related to Figure 2. (**A**) Scheme for set-ups used to study (1.) CAR immune effector cell tumor infiltration using 3D confocal imaging, (2.) CAR immune effector cell persistence using flow cytometry and (3.) the effect of CAR immune effector cell therapy on the tumor microenvironment using scRNA-seq. (**B-D**) GL-261 iRFP720 glioma-bearing C57BL/6 received intravenous injections of 5 x 10^6 CAR immune effector cells co-expressing ZsGreen on day 11 after glioma cell implantation. Mice were perfused two days later, and brain sections stained for DAPI and the endothelial cell marker endomucin. Images were processed and evaluated using Imaris. CDF plots of distances to the endothelium are shown in red compared to simulated complete spatial random (CSR) distributions in grey for CAR T cells (**B**), CAR NK cells (**C**) and CAR macrophages (**D**). Two-sample Kolmogorov–Smirnov test was used to analyze significance (CDF versus CSR plots: $^*P < 0.05$).

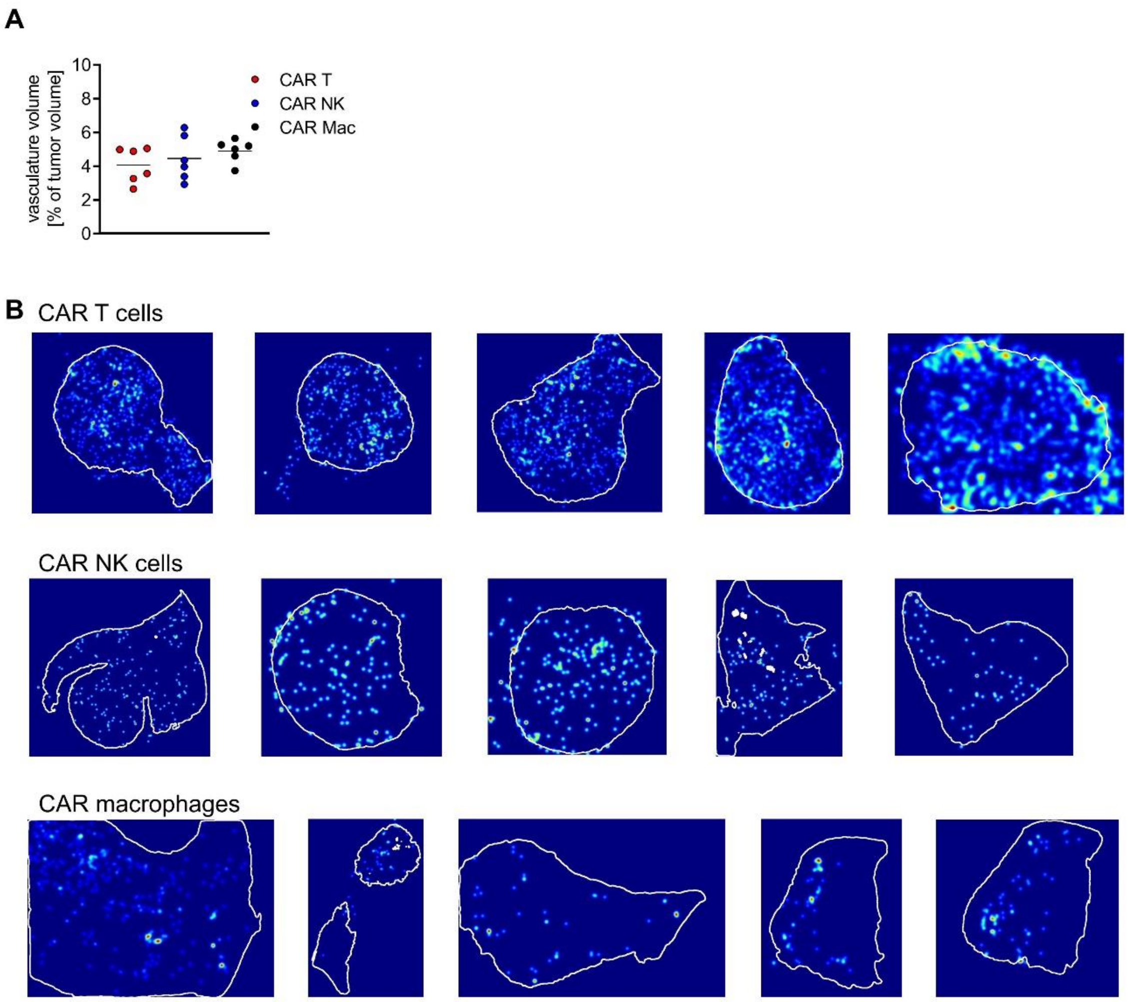

Figure S4: Spatial distribution of CAR immune effector cells within the tumor and consequences on the tumor size. Related to Figure 2. (**A-B**) GL-261 iRFP720 glioma-bearing C57BL/6 received intravenous injections of 5 x 10^6 CAR immune effector cells co-expressing ZsGreen on day 11 after glioma cell implantation. Mice were perfused two days later and brain sections stained for DAPI and the endothelial cell marker endomucin. Images were processed and evaluated using Imaris. (**A**) Quantification of relative vasculature volume per tumor volume and (**B**) 2D tissue maps of CAR T cell, CAR NK cell and CAR macrophages within whole tumor sections of different mice are shown.

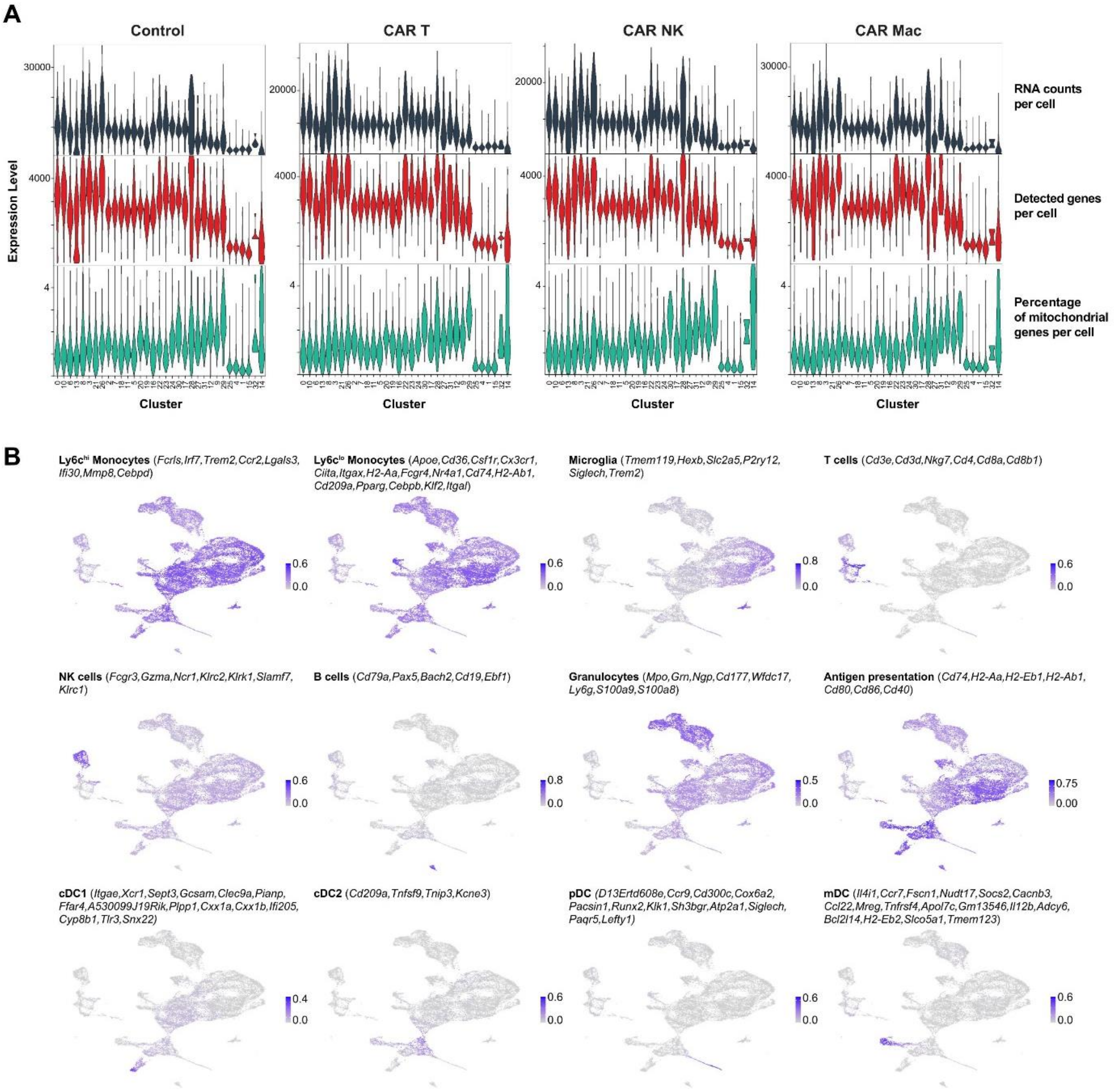

Figure S5: High quality single-cell RNA-sequencing data identifies cell types via their transcriptional modules. Related to Figure 3. GL-261 glioma cells were implanted orthotopically into the brain of C57BL/6 wild-type mice. On day 7 after implantation 2 x 10^6 CAR T cells, CAR NK cells or CAR macrophages were injected intratumorally and 5 days later the tumor isolated and dissociated. $CD45^+$ immune cells were FACS sorted and subjected to scRNA-seq. (**A**) Clusterwise violin plot visualization of the quality control metrics across the treatment groups. (**B**) UMAPs color-coded for the expression of the indicated transcriptional modules.

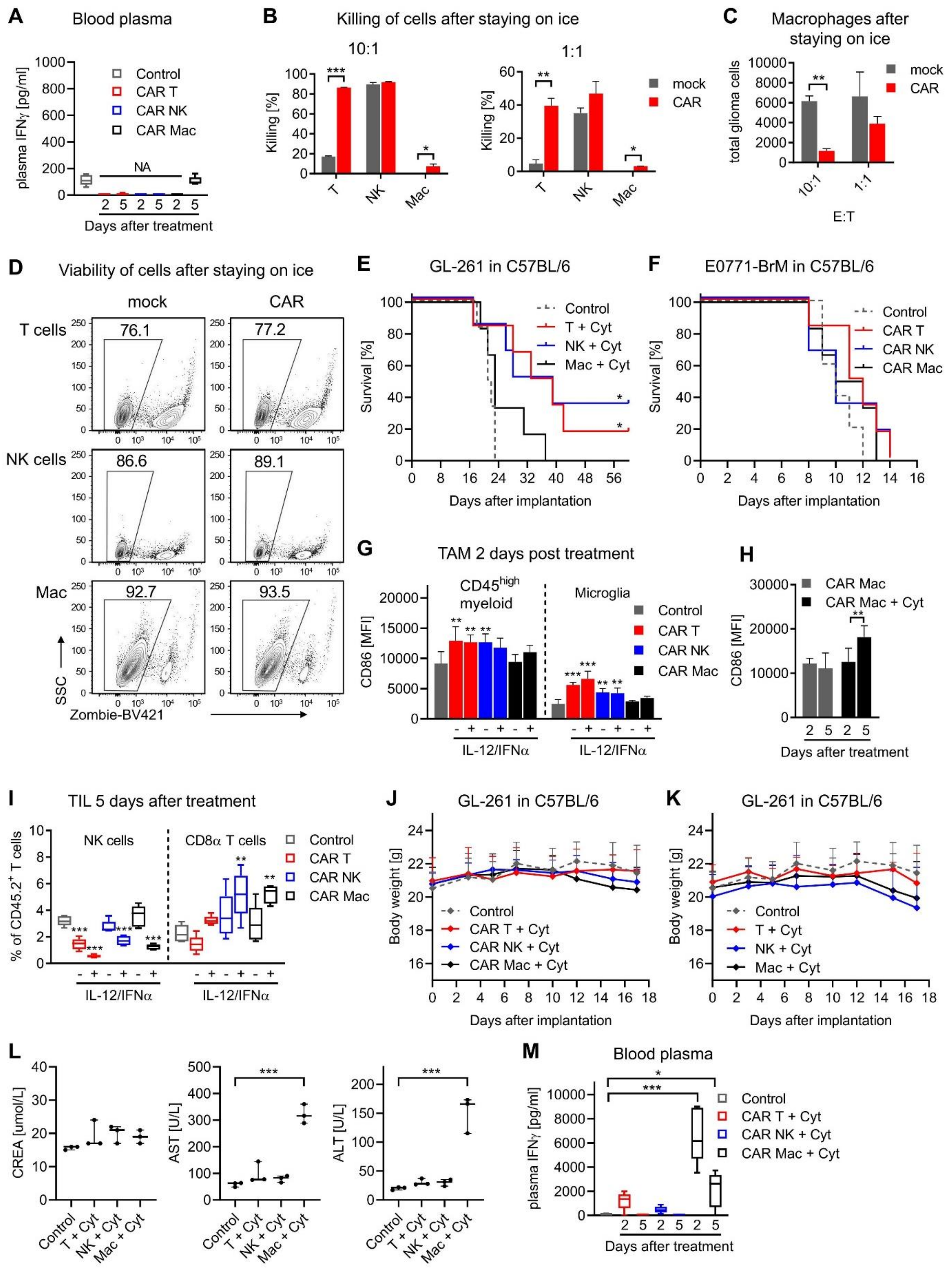

Figure S6: Mouse immune cells remain effector functions on ice and prove safe and efficient if co-expressing pro-inflammatory cytokines in vivo. Related to Figure 4 and 5. (**A**) GL-261 glioma cells were implanted orthotopically in C57BL/6 wild-type mice. Mice received intratumoral treatment with 2 x 10^6 CAR

immune effector cells 7 days after tumor implantation and blood plasma was collected 2 or 5 days later to quantify IFNγ concentration using ELISA (boxplot with median +/- quartiles and min to max of $n = 5$, one-way ANOVA with *$P < 0.05$; **$P < 0.01$, ***$P < 0.001$.) (**B-D**) Mouse T cells, NK cells and macrophages were mock transfected or transfected with CAR encoding mRNA and analyzed after staying on ice for adoptive cell therapy. (**B**) Cell lysis and (**C**) total cell counts of SB-28 glioma cells that were co-cultured at an E:T ratio of 10:1 or 1:1 for 24 hours with mouse immune cells (mean + SD of $n = 3$, one-way ANOVA with *$P < 0.05$; **$P < 0.01$; ***$P < 0.001$). (**D**) Cell viability was assessed using flow cytometry. (**E**) GL-261 glioma cells or (**F**) E0771-BrM breast cancer cells were implanted orthotopically into the brain of C57BL/6 wild-type mice. Mice received intratumoral treatment with either CAR mRNA transfected immune cells or IL-12 and IFNα2 (Cyt) mRNA transfected immune cells on day 5 and day 10 after tumor implantation. Survival data of $n = 6$ mice per treatment group are presented as Kaplan-Meier plots. P values were calculated with log-rank test (treatment versus control: *$P < 0.05$). (**G-I**) GL-261 glioma cells were implanted orthotopically in C57BL/6 wild-type mice. Mice received intratumoral treatment with 2 x 10^6 CAR immune effector cells or 2 x 10^6 multifunctional CAR immune effector cells 7 days after tumor implantation and tumors were collected 2 or 5 days later to perform flow cytometry on tumor-infiltrating immune cells. (**G**) CD86 surface expression on tumor infiltrating $CD45^{high}$ myeloid cells and microglia (mean + SD of $n = 5$, one-way ANOVA with *$P < 0.05$; **$P < 0.01$, ***$P < 0.001$.). (**H**) CD86 surface expression on adoptively transferred (multifunctional) CAR macrophages 2 or 5 days after intratumoral injection (mean + SD of $n = 5$, unpaired t test with *$P < 0.05$; **$P < 0.01$, ***$P < 0.001$). (**I**) Relative abundance of tumor infiltrating NK cells and CD8α T cells (boxplot with median +/- quartiles and min to max of $n = 5$, one-way ANOVA with *$P < 0.05$; **$P < 0.01$, ***$P < 0.001$.) (**J-K**) Bodyweight curves of glioma-bearing mice that received intratumoral treatment with either multifunctional CAR immune effector cells (**J**) or IL-12 and IFNα2 (Cyt) expressing immune effector cells (**K**) on day 5 and day 10 after tumor implantation over the course of treatment (mean + SD of $n = 6$). (**L**) Blood of mice from E was collected 12 days after tumor cell implantation. Blood of two mice each was pooled and clinical parameters (CREA, AST, ALT) were analyzed (boxplot with median +/- quartiles and min to max of $n = 3$, one-way ANOVA with *$P < 0.05$; **$P < 0.01$; ***$P < 0.001$). SSC = Side Scatter; CREA, creatinine; AST, aspartate aminotransferase; ALT, alanine transaminase. (**M**) Same setup as in A. Mice received intratumoral treatment with 2 x 10^6 multifunctional CAR immune effector cells 7 days after tumor implantation and blood plasma was collected 2 or 5 days later to quantify IFNγ concentration using ELISA (boxplot with median +/- quartiles and min to max of $n = 5$, one-way ANOVA with *$P < 0.05$; **$P < 0.01$, ***$P < 0.001$.)

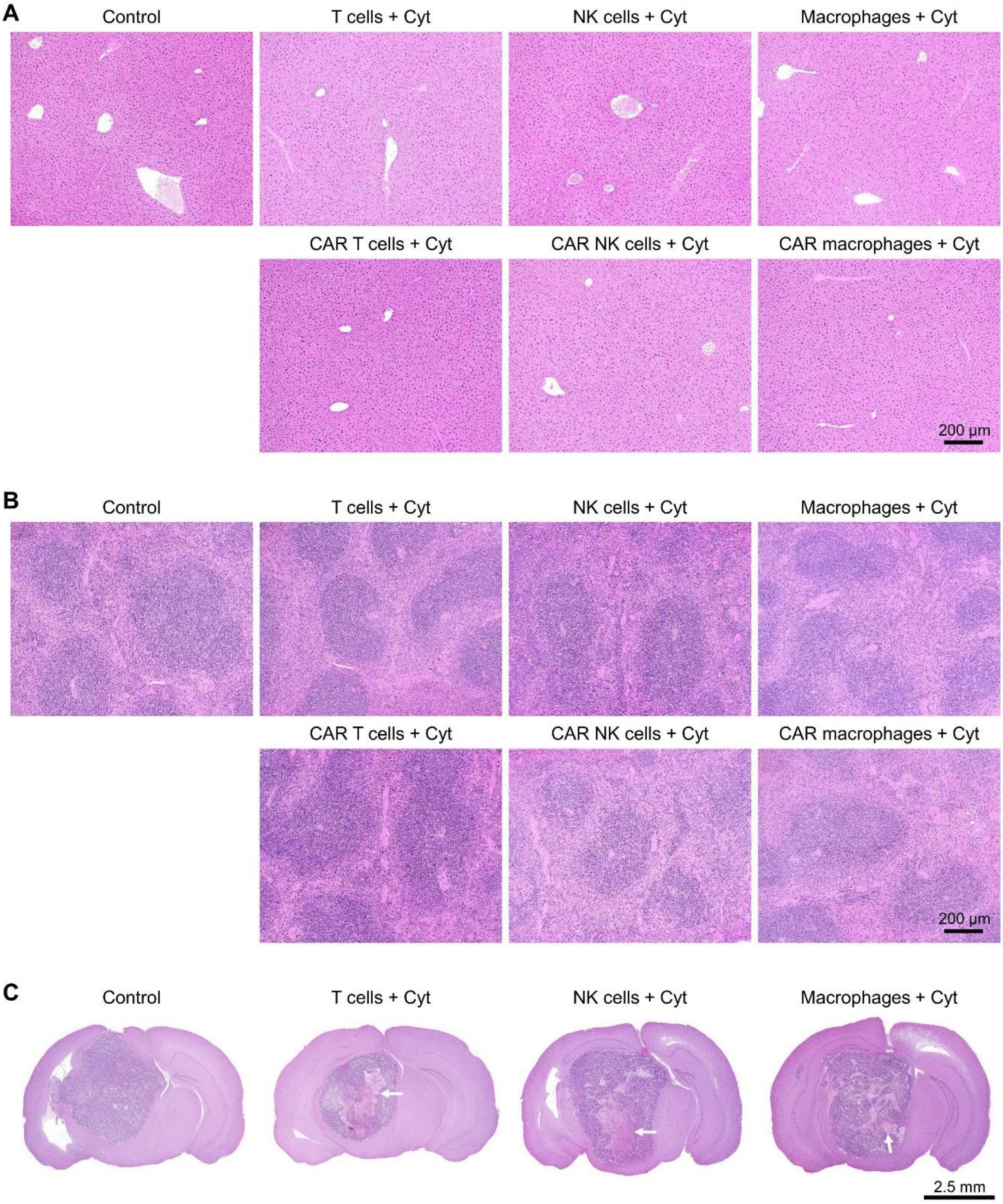

Figure S7: H&E images of organs from CAR immune effector cell treated mice. Related to Figure 5. Representative pictures of H&E-stained sections of mouse liver (**A**) and spleen at 10x magnification (**B**) and glioma-bearing mouse brains at 1.25x magnification (**C**). The white arrow indicates areas of necrosis.

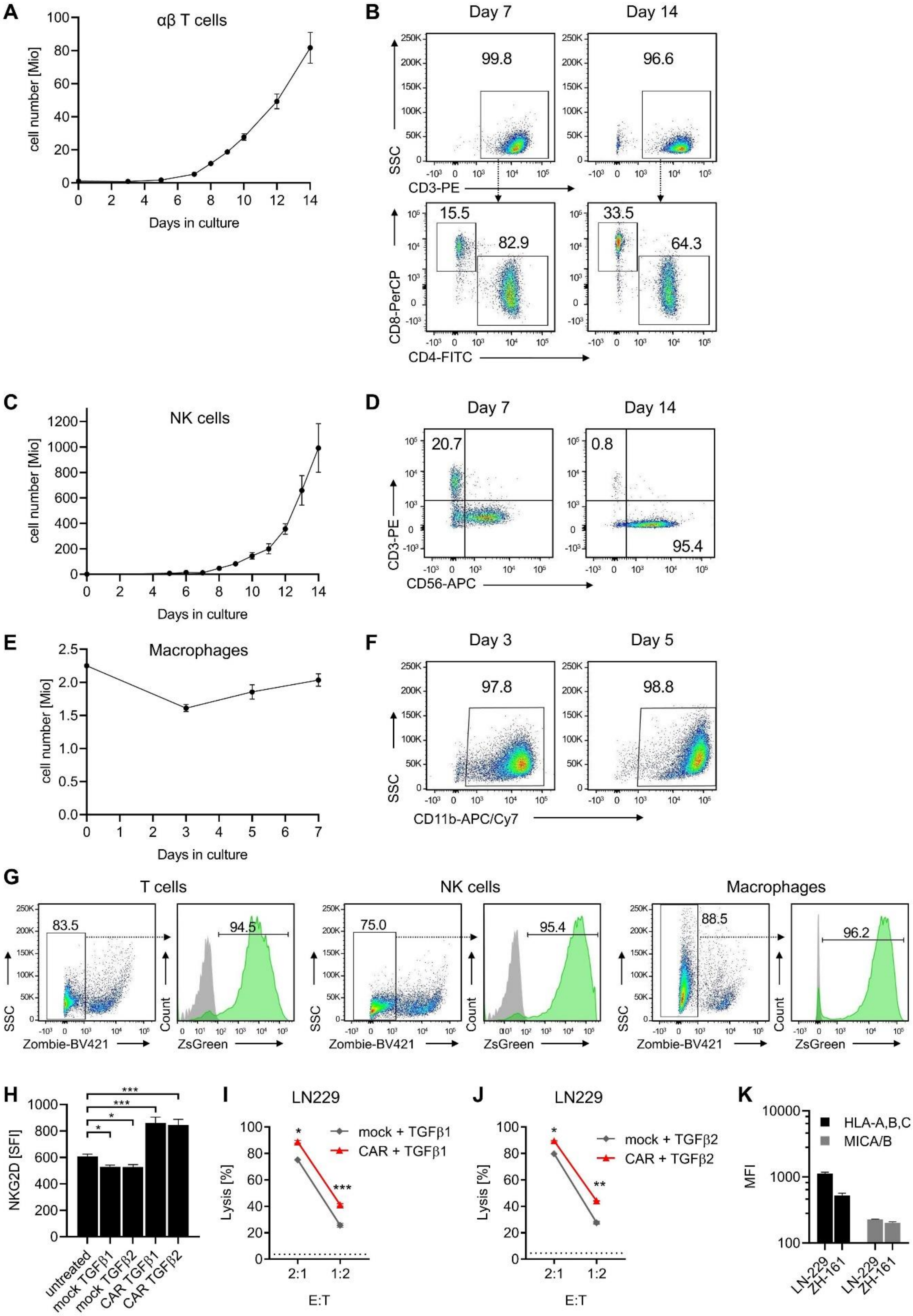
A
αβ T cells
cell number [Mio]
Days in culture
B
Day 7
Day 14
99.8
96.6
SSC
CD3-PE
15.5
82.9
33.5
64.3
CD8-PerCP
CD4-FITC
C
NK cells
cell number [Mio]
Days in culture
D
Day 7
Day 14
20.7
0.8
95.4
CD3-PE
CD56-APC
E
Macrophages
cell number [Mio]
Days in culture
F
Day 3
Day 5
97.8
98.8
SSC
CD11b-APC/Cy7
G
T cells
NK cells
Macrophages
83.5
94.5
75.0
95.4
88.5
96.2
SSC
Count
Zombie-BV421
ZsGreen
H
NKG2D [SFI]
untreated
mock TGFβ1
mock TGFβ2
CAR TGFβ1
CAR TGFβ2
I
LN229
mock + TGFβ1
CAR + TGFβ1
Lysis [%]
E:T
J
LN229
mock + TGFβ2
CAR + TGFβ2
Lysis [%]
E:T
K
HLA-A,B,C
MICA/B
MFI
LN-229
ZH-161

Figure S8: Human immune effector cell expansion from PBMCs and mRNA transfection in vitro. Related to Figure 6. (**A, B**) Human T cells were isolated from PBMCs using CD3+ magnetic beads, activated using CD3/CD28 activation beads and kept in medium supplemented with 100 U/ml IL-2. (**C, D**) Human NK cells were negatively selected from PBMCs using CD3- magnetic beads, co-cultured with irradiated (100 Gy) K562-mbIL21 feeder cells and medium supplemented with 200 U/ml IL-2. Negative selection and co-culture were repeated weekly. (**E, F**) Human macrophages were isolated from PBMCs using CD14+ magnetic beads and kept in medium supplemented with 50 ng/ml M-CSF. (**A, C, E**) Proliferation curves and (**B, D, F**) cell characterization using flow cytometry during expansion are shown (mean +/- SD of $n = 3$). (**G**) Human T cells, NK cells and macrophages were electroporated with ZsGreen mRNA. Cell viability and ZsGreen fluorescence were assessed 24 later using flow cytometry. SSC = Side Scatter. (**H-J**) Mock or CAR mRNA-transfected human NK cells were cultured alone or together with LN-229 glioma cells in the presence of 50 ng/ml TGFβ1 or TGFβ2. (**H**) Flow cytometry quantification of NKG2D surface expression on NK cells 12 hours after incubation with TGFβ1 or TGFβ2 (mean + SD of $n = 3$, one-way ANOVA with *$P < 0.05$; **$P < 0.01$; ***$P < 0.001$). (**I-J**) Glioma cell lysis after co-culture (mean +/- SD of $n = 3$, paired t test with *$P < 0.05$; **$P < 0.01$, ***$P < 0.001$). (**K**) Flow cytometry quantification of absolute HLA-A,B,C and MICA/B surface expression on LN-229 and ZH-161 glioma cells are shown (mean + SD of $n = 3$).

Table S1. Overview of mRNAs used for immune cell electroporation. Related to Figure STAR METHODS. Indicated are mRNA concentrations used for transfection in 100 µl.

mRNA	Amount per 100 µl electroporation reaction
mouse/human CAR	10 ug
mouse/human CARΔ(CD3ζ)	10 ug
mouse/human IL12	2.5 ug
mouse/human IFNα2	2.5 ug
ZsGreen (in vitro)	2.5 ug
ZsGreen (in vivo)	5 ug
