# Supplementary material for: CAR T cells, CAR NK cells, and CAR macrophages exhibit distinct traits in glioma models but are similarly enhanced when combined with cytokines
Source: Cell Rep Med. 2025 Jan 30;6(2):101931. doi: 10.1016/j.xcrm.2025.101931 (PMC11866521; doi:10.1016/j.xcrm.2025.101931)
Supplement: Data S1. Consent form, related to Figure 6 [file mmc2.pdf]

Information sheet

Further use of health-related personal data and biological material for research purposes

Version 3.0 of 01/10/2019, en

Dear Patient

During your stay at University Hospital Zurich (USZ), health-related data and possibly also biological material from your body (samples of blood and other bodily fluids, tissue samples) will be gathered from you.
This biological material in connection with the data is also very valuable for medical research. We therefore ask for your consent to use this material and your data for research purposes.

Your consent for research purposes

With your consent, researchers can scientifically analyse your data and conduct tests on samples that have been taken from you. This consent concerns data at our hospital that relate to your health or person. This includes entries in your medical history concerning the course of disease and treatments you have received, results of imaging examinations or laboratory testing, details of your genetic predisposition to certain illnesses (genetic data) and details about your person (age, gender).
The samples involve previously removed biological material (blood, urine or tissue) that is no longer required for the purposes of diagnosis or treatment.

It is possible that during the course of your stay at our hospital you will be asked again to provide consent for research purposes. This may be the case if the clinic that is responsible for you wishes to take additional samples from you or scientifically examine a specific issue.

USZ Universitäts Spital Zürich

Please note the information on the reverse

A4_Generalkonsent_englisch.indd 1 05.12.19 17:03

Protection of your data and samples
The use of the data and samples and their forwarding to researchers in Switzerland and abroad are subject to strict regulations. Only a small number of people are authorised to view your medical history. These people are responsible for your treatment or have permission within the framework of a research project to view your data.

Data used for research purposes must be **coded** as swiftly as possible, meaning that all identifying details – such as your name, date of birth, insurance number, etc. – are replaced by a code. Only people with access to the key (a document matching codes and names) can thus associate them with your person.

The **samples (biological material)** are stored securely in a biobank. A biobank is a systematic collection of samples and associable data stored under clearly stipulated conditions. Samples and genetic data may only be passed on to researchers if they are **coded or** anonymised. Anonymised means that all the identifying details have either been rendered unrecognisable or deleted so that it is no longer possible to trace them back to your person.

Forwarding of your data and samples
If data and samples are forwarded in coded form to researchers **outside** University Hospital Zurich, the key will remain at USZ, where it will be stored securely by an office not involved in the research project. For research conducted abroad, it must be ensured that at least the same data protection requirements are upheld that apply in Switzerland.

In general, research projects must also be approved by the local ethics committee. This assesses whether the project and its conduct are scientifically and ethically sound, and whether the legal requirements, in particular data protection, are complied with.

Research results
The findings from research projects involving data and samples usually contribute to improved medical care only for future patients. If, however, certain results should prove relevant for your own health, you will be informed of this as far as possible (this is not feasible in research with anonymised samples). However, such situations occur very rarely.

By volunteering your data and samples for research purposes, you waive the right to any share in possible profits that could arise from the results. Neither you nor your health insurer will incur **any costs** resulting from the research projects.

Your rights
Your consent is voluntary and generally has no expiration. However, you are entitled at any time to withdraw your consent without stating the reasons **(revocation)**. To do so, please contact the clinic treating you. In the event of revocation your data and samples will no longer be made available for researchprojects.

Deciding for or against granting your consent and withdrawing your consent will have no effect on your medical car.

→ **Should you have any other questions about the further use of your data and samples for research purposes, please contact the doctor treating you or visit our website www.en.usz.ch/research**

Declaration of consent

Patient label

to the further use of health-related personal data and biological material for research purposes

Last name and first name of patient:

Date of birth:

I confirm that

– I have received the information sheet that is part of this declaration of consent, and feel sufficiently informed.

I consent to

– the further use of my health-related data (incl. genetic data) and biological material as described above for research purposes.

☐ **Yes**

☐ **No**

By permitting the use of your health-related data and samples, you are making a valuable contribution to biomedical research.

Thank you very much for this.

General consent, en, version 3.0 of 01/10/2019

Town/city

Date __ __ . __ __ . __ __ __ __

Patient signature

Only if a minor or without legal capacity:
Signature of authorised representative
